# Molecular Mechanisms and Metabolic Responses in the Biological Antagonism Between *Trichoderma harzianum* and *Fusarium oxysporum*

**DOI:** 10.3390/microorganisms14051068

**Published:** 2026-05-09

**Authors:** Taozhen Chen, Keyang Tao, Yanguang Zhou, Hao Qian, Binchao Yu, Mingjiang Mao, Chendao Ruan, Xiaofeng Yuan

**Affiliations:** 1 School of Life Sciences, Zhejiang Chinese Medical University, Hangzhou 310053, China; chentaozhen@zcmu.edu.cn (T.C.); 13758562975@163.com (K.T.); zhouyanguang07@163.com (Y.Z.); qianhao0@foxmail.com (H.Q.); a2930205638@163.com (B.Y.); zcmummj2021@163.com (M.M.); 2 State Key Laboratory of Soil Pollution Control and Safety, Zhejiang University, Hangzhou 310058, China; 12514001@zju.edu.cn

**Keywords:** *Trichoderma harzianum*, *Fusarium oxysporum*, metabolomics, transcriptomics, mycotoxins, RNAi

## Abstract

*Trichoderma harzianum* is an important biocontrol fungus widely used to manage vascular wilt caused by *Fusarium oxysporum*. However, the molecular regulation and metabolic responses underlying different confrontation modes remain unclear. In this study, we integrated non-targeted and targeted metabolomics, transcriptomics, mycotoxin detoxification assays, and RNA interference (RNAi) to systematically investigate antagonistic mechanisms under direct and indirect confrontation conditions. Direct confrontation strongly inhibited *F. oxysporum* via physical mycoparasitism and was accompanied by enhanced mycotoxin biosynthesis. In contrast, indirect confrontation induced metabolic reprogramming, characterized by increased amino acid and energy metabolism, and promoted biomass accumulation in *T. harzianum*. Targeted metabolomics identified 38 core mycotoxins, several of which were significantly enriched during direct confrontation. Detoxification assays further showed that *T. harzianum* reduced multiple mycotoxins in a concentration-dependent manner, likely through a combination of physical adsorption and potential biochemical transformation, although the exact mechanisms remain unclear. Transcriptomic analysis revealed extensive differential gene expression in both fungi, particularly in pathways related to redox homeostasis and metabolic regulation. In addition, exogenous dsRNA effectively reduced the expression of selected pathogenicity-related genes in *F. oxysporum* at the transcriptional level. Overall, these findings highlight mode-specific antagonistic responses and provide a descriptive framework for understanding fungal interactions. The potential integration of microbial biocontrol with RNAi-based approaches is discussed as a conceptual perspective that requires further experimental validation.

## 1. Introduction

*Fusarium oxysporum* is a plant pathogen with a broad host range that infects various crops, leading to vascular wilt and significant economic losses worldwide [1]. Biological control based on beneficial fungi has emerged as an effective and sustainable strategy for managing plant diseases. Among these, *Trichoderma harzianum* has attracted considerable interest owing to its broad-spectrum antagonistic activity and strong biocontrol performance [2]. Previous studies have demonstrated that *T. harzianum* inhibits pathogenic fungi through multiple mechanisms, including the secretion of cell wall-degrading enzymes, the production of antifungal secondary metabolites, and cometition for nutrients and ecological niches [3,4,5].

However, microbial interactions are not limited to direct physical contact but also involve intricate metabolic exchanges and chemical communication [6]. Under different confrontation conditions, the stress responses, metabolic reprogramming, and regulation of mycotoxin biosynthesis in *F. oxysporum* remain poorly understood. In particular, the distinct effects of direct contact compared with volatile-mediated interactions have not yet been systematically evaluated [7].

In recent years, notable advances have been achieved in understanding these processes. For instance, *F. oxysporum* can activate polyketide synthase gene clusters under stress conditions to produce red pigments, which may enhance stress tolerance [8]. Antagonistic microbial interactions can influence mycotoxin profiles [9]. Recent investigations have also emphasized the roles of volatile compounds and direct mycoparasitism in modulating reactive oxygen species (ROS) and energy metabolism [10]. Nevertheless, the mode-specific regulation of mycotoxin biosynthesis pathways and the association between pigment formation and toxin accumulation remain unclear.

Beyond inhibiting fungal growth, *T. harzianum* can also mitigate mycotoxin contamination. This effect may occur either indirectly by restricting toxin accumulation or directly through biodegradation into less toxic derivatives [11,12]. However, most existing studies have concentrated on the degradation efficiency of individual toxins, such as aflatoxin B1 (AFB1) [13]. Comprehensive studies addressing concentration-dependent effects, environmental influences, and the combined contributions of physical adsorption and enzymatic transformation are still limited. This limitation is particularly evident for *Fusarium* mycotoxins such as T-2 toxin (T-2) and zearalenone (ZEN) [14,15].

RNA interference (RNAi) has recently emerged as a promising approach for precise disease control. Exogenous dsRNA can silence key genes associated with pathogenicity in *F. oxysporum*, thereby reducing its virulence and potentially enhancing the efficacy of *Trichoderma*-mediated biocontrol [16,17,18]. Advances in nanodelivery platforms and spray-induced gene silencing (SIGS) have further expanded the practical applications of RNAi. However, challenges persist in the large-scale production of dsRNA and in effectively integrating RNAi with established biocontrol strategies [19,20,21].

Although numerous studies have confirmed the effectiveness of *T. harzianum* against Fusarium wilt, most have emphasized overall antagonistic performance or individual mechanisms, such as mycoparasitism, nutrient competition, and antifungal metabolite production [22,23]. While some studies have reported successful applications of *Trichoderma* species in agricultural systems, these investigations mainly focus on phenotypic outcomes or single-level analyses [24]. Integrated studies that combine metabolic and transcriptional responses of both organisms during antagonistic interactions remain limited.

In particular, how different confrontation modes—direct contact versus indirect volatile-mediated interaction—affect metabolic responses, toxin biosynthesis, and regulatory networks in *F. oxysporum* has not been systematically explored. Furthermore, the mechanisms responsible for mycotoxin detoxification by *Trichoderma* and their responses to environmental factors remain insufficiently clarified [14]. At the same time, although RNAi shows strong potential for pathogen control, its integration with microbial biocontrol systems has been rarely investigated.

Rather than focusing on individual mechanisms, this study aims to comparatively dissect how different confrontation modes (direct versus indirect) reshape metabolic and transcriptional responses in both organisms within a unified experimental framework. Importantly, this work does not seek to establish definitive molecular mechanisms, but instead to identify mode-dependent patterns and potential regulatory hubs that may guide future mechanistic studies. In addition, although RNAi is explored as a potential complementary strategy, its integration with microbial biocontrol is presented here as a preliminary and exploratory concept rather than a validated application.

## 2. Materials and Methods

### 2.1. Main Reagents

Cellophane (AC17349, Xiamen Huiyao, Xiamen, China) and CNW QuEChERS cleanup cartridges (SBEQ-CA8805-H, Anpel, Shanghai, China) were used for sample preparation. RevertAid First Strand cDNA Synthesis Kit (K1622), dNTPs (AM8200) were purchased from Thermo Fisher Scientific (Waltham, MA, USA). UNlQ-10 Column TRIzol Total RNA Isolation Kit (B511321) and 2× TaqMan Fast qPCR Master Mix (B639274) were obtained from Sangon Biotech (Shanghai, China). QuickCut™ restriction enzymes including Nde I, Xho I, Bgl II and Kpn I (1621, 1635, 1606 and 1618) were purchased from TaKaRa (Kusatsu, Japan). *Escherichia coli* DH5α (harboring L4440 plasmid) and HT115(DE3) strains were obtained from BNCC (Shanghai, China). RNA Extraction Agent (ET121) was purchased from TransGen Biotech (Beijing, China).

### 2.2. Experimental Methods

#### 2.2.1. Cultivation of *F. oxysporum* and *T. harzianum*

The fungal strains used in this study were *F. oxysporum* (BNCC120618) and *T. harzianum* (BNCC336568). Both strains were obtained from the Beijing Biochemical Culture Collection Center (BNCC, Beijing, China) via commercial distributors. Species identity was confirmed by sequencing the internal transcribed spacer (ITS) region. The sequences were deposited in the NCBI GenBank database under accession numbers PZ210310 (*F. oxysporum*) and PZ210309 (*T. harzianum*).

Three experimental conditions were established: control, direct confrontation, and indirect confrontation, each with three biological replicates.

In the control group, *F. oxysporum* and *T. harzianum* were cultured separately and designated as cFO and cTH, respectively.

For direct confrontation, the two fungi were co-cultured on PDA plates covered with cellophane. After incubation, mycelia of each fungus were collected separately from the same plate and designated as dFO and dTH. The inhibition rate was calculated based on biomass as follows: inhibition rate (%) = (MC − MT)/MC × 100.

For indirect confrontation, a non-contact co-culture system was used to assess the effects of volatile compounds produced by *T. harzianum*. Mycelia of both fungi were collected separately and designated as vFO and vTH. Inhibition rates were calculated as described above.

#### 2.2.2. Non-Targeted Metabolomics Analysis of Metabolic Products in Dual-Fungal Confrontation

Metabolites were extracted following the method described by Pu et al. [25]. Mycelia were extracted with 80% methanol containing internal standards, followed by repeated freeze–thaw cycles in liquid nitrogen, low-temperature centrifugation, and impurity precipitation. The resulting supernatant was subjected to LC–MS analysis.

Chromatographic separation and mass spectrometric detection were performed as previously described [26], using a Waters ACQUITY Premier HSS T3 column (Waters, Milford, MA, USA) coupled with an AB TripleTOF 6600 mass spectrometer (AB SCIEX, Framingham, MA, USA) operating in both ESI^+^ and ESI^−^ modes. Water and acetonitrile containing 0.1% formic acid were used as the mobile phases. The flow rate was 0.4 mL min^−1^, and the injection volume was 4 μL (Table S1).

Raw data were converted to mzXML format using ProteoWizard (version 3.0.20233) and processed with XCMS for peak detection, alignment, and retention time correction. Features with a missing rate >50% across samples were removed, and remaining missing values were imputed using the k-nearest neighbor (KNN) method. Peak areas were normalized using support vector regression (SVR).

Metabolite annotation was performed by matching against an in-house database, public databases, predictive libraries, and the metDNA approach. Only metabolites with an identification score ≥ 0.5 and a coefficient of variation (CV) < 0.3 in quality control (QC) samples were retained. For metabolites detected in both ionization modes, the one with the higher identification level and lower CV was selected for further analysis.

#### 2.2.3. Targeted Metabolomics Analysis of *F. oxysporum* Mycotoxins

Following the method described by Kolawole et al. [27], an in-house database of *F. oxysporum* mycotoxins was constructed using Compound Discoverer 3.3. Samples were purified using CNW QuEChERS cleanup cartridges, evaporated under reduced pressure, and reconstituted in 80% methanol–water prior to LC–MS analysis.

Targeted LC–MS analysis was performed on a C18 analytical column coupled with an Orbitrap high-resolution mass spectrometer. The mobile phases consisted of 0.1% formic acid in water (A) and acetonitrile (B), using gradient elution. The flow rate was 1 mL min^−1^, and the injection volume was 10 μL (Table S1).

Raw data were processed using Compound Discoverer 3.3 for peak extraction, alignment, and retention time correction. Missing values were imputed using the KNN method, followed by SVR for peak area normalization.

Metabolites were identified by matching against the in-house mycotoxin database. All targeted metabolites were confirmed using authentic standards. Targeted metabolomics analysis was performed by MWMETABOL (Wuhan, China).

#### 2.2.4. Determination of *T. harzianum* Degradation Capacity for *F. oxysporum* Mycotoxins

Standard toxins (AFB1, T-2, and ZEN) were initially dissolved in benzene–acetonitrile (97:3, *v*/*v*), evaporated under nitrogen, redissolved in methanol, and subsequently diluted in PBS. To determine saturation concentrations for toxin binding, 1 × 10^8^ CFU/mL *T. harzianum* cells were co-incubated with each toxin at concentrations of 300, 200, 100, 50, 10, and 1 μg/mL for 24 h. Following centrifugation, the supernatant was analyzed by HPLC to quantify residual toxin concentrations.

For time-course binding assays, fungal cells were incubated with toxin solutions at saturation concentrations at 27 °C for different time intervals. After centrifugation, residual toxin levels in the supernatant were measured by HPLC. Binding rates were calculated as (C_0_ − C_t_)/C_0_ × 100%, where C_0_ represents the initial toxin concentration and C_t_ represents the toxin concentration at time t.

AFB1 was quantified using fluorescence detection with a mobile phase of water–acetonitrile–methanol (6:30:10, *v*/*v*/*v*) at excitation and emission wavelengths of 365 and 435 nm, respectively. T-2 toxin was quantified by UV detection using acetonitrile–water (60:40, *v*/*v*) at 212 nm, while ZEN was quantified using acetonitrile–0.1% formic acid water (5:95, *v*/*v*) at 274 nm. Toxin concentrations were determined using standard curves (Figure S1). All experiments were performed in triplicate.

#### 2.2.5. Stability Study of *T. harzianum*-Bound *F. oxysporum* Mycotoxin Complexes

After toxin binding, mycelia were washed five times with ultrapure water, and toxin release in each wash was quantified by HPLC. The effects of pH and temperature on binding stability were subsequently evaluated. Using the five pure-water washes as the control, three additional stress treatments were applied to assess binding stability under extreme conditions: high-pressure sterilization (121 °C for 40 min), ultrasonication in an ice-water bath for 40 min, and chloroform extraction with oscillation followed by redissolution. Released toxins in post-treatment solutions were quantified by HPLC. Data were analyzed using GraphPad Prism software (version 10.1.2). Statistical significance was evaluated by one-way ANOVA followed by Tukey’s multiple comparison test. Differences in complex stability were assessed using Student’s *t*-test, with *p* < 0.05 considered statistically significant.

#### 2.2.6. Transcriptomics Analysis

Total RNA was extracted using the CTAB-PBIOZOL method followed by ethanol precipitation. RNA quality was assessed prior to library construction.

For transcriptome sequencing, mRNA was fragmented and reverse-transcribed into first-strand cDNA, followed by synthesis of double-stranded cDNA. The resulting cDNA underwent end repair, dA-tailing, adapter ligation, fragment size selection, and PCR amplification to generate sequencing libraries. Paired-end sequencing was performed on the Illumina platform.

Raw sequencing data were quality-filtered using fastp 1.0, followed by de novo transcriptome assembly with Trinity 2.15.2. Redundant transcripts were clustered and removed using Corset 1.06, and coding sequences (CDSs) were predicted with TransDecoder (version 5.7.1).

Functional annotation was performed by aligning transcripts against multiple databases, including KEGG, NR, and GO, using Diamond 5.1.1 and HMMER 3.3.2. Gene expression levels were quantified as fragments per kilobase of transcript per million mapped reads (FPKM) using RSEM 1.3.3. Differentially expressed genes were identified using DESeq2 1.48.1, followed by KEGG and GO enrichment analyses.

Hypothetical proteins and conserved domains were further validated through integrated analysis of KEGG annotations, BLAST 2.15.0 alignment against the *T. harzianum* genome, and searches against the Pfam and Conserved Domain Database (CDD).

#### 2.2.7. RT-qPCR Validation of Interaction-Related Genes

Total RNA was extracted from the cFO, dFO, and vFO groups. First-strand cDNA was synthesized using the reverse transcription kit under the following conditions: 25 °C for 5 min, 42 °C for 60 min, and 70 °C for 5 min. A 20 μL quantitative PCR (qPCR) reaction mixture was prepared using SYBR Green premix, and amplification was performed with gene-specific primers listed in Table S2.

#### 2.2.8. dsRNA Design and Functional Validation

Target genes in *F. oxysporum* (including *FOXG_09492* and *FOXG_00252*) were selected based on transcriptomic analysis and previous studies [28]. dsRNA sequences were designed according to target sequence characteristics, predicted secondary structure, and off-target potential (Table S3), with detailed design parameters provided in Table S4. Potential off-target effects were evaluated by BLAST alignment against the *F. oxysporum* genome, and dsRNA fragments with minimal homology to non-target genes were selected.

Templates were amplified using primers containing T7 promoter sequences, followed by in vitro transcription for dsRNA synthesis. The resulting dsRNA products were purified, enzymatically treated, and verified by agarose gel electrophoresis.

Mycelia in the logarithmic growth phase (OD_600_ ≈ 0.6) were treated with 50 μg/mL dsRNA in culture medium for 12 h, with sterile water serving as the control. After treatment, samples were rapidly frozen in liquid nitrogen, and target gene expression was analyzed by RT-qPCR.

Although sequence alignment was used to reduce potential off-target effects, no non-specific dsRNA control was included. Therefore, the specificity of the observed gene-silencing effects should be interpreted with caution.

#### 2.2.9. Rapid dsRNA Preparation Using *Escherichia coli*

Target gene fragments were amplified from *F. oxysporum* cDNA using primers containing *Kpn I* and *Bgl II* restriction sites (Table S2). After purification, the amplified fragments were ligated into the similarly double-digested L4440 vector to generate recombinant plasmids (Figure S2).

The recombinant plasmids were transformed into *Escherichia coli HT115* competent cells prepared using the CaCl_2_ method. Positive transformants were screened by ampicillin resistance and confirmed by PCR. dsRNA was subsequently extracted using two different methods for comparison. The TRIzol method involved bead-beating disruption, TransZol extraction, phase separation, and precipitation. The ethanol fixation method involved fixation in 75% ethanol, resuspension in NaCl solution, and centrifugation to recover the supernatant. The efficiency of the two dsRNA extraction methods was compared in subsequent analyses.

#### 2.2.10. Data Processing

All experiments were conducted with three independent biological replicates. Data are presented as mean ± standard deviation (SD), unless otherwise specified. Relative gene expression levels were calculated using the 2^−ΔΔCT^ method. Statistical analyses were performed using SPSS 20.0 software with one-way analysis of variance (ANOVA). Graphs were generated using GraphPad Prism. Significant differences among groups were determined at *p* < 0.05 and are indicated by different letters.

## 3. Results

### 3.1. Differential Effects of Confrontation Modes on Fungal Growth and Phenotypic Responses

**Figure 1:**
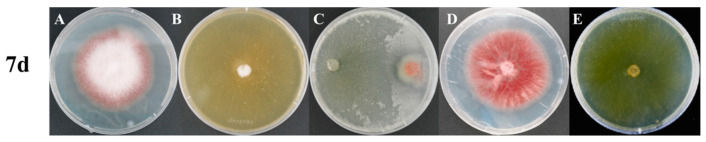
Phenotypic changes during fungal confrontation after 7 days of incubation. (**A**) Monoculture of *F. oxysporum* (cFO); (**B**) monoculture of *T. harzianum* (cTH); (**C**) direct confrontation between *F. oxysporum* and *T. harzianum*, with *T. harzianum* positioned on the left and *F. oxysporum* on the right (dFO and dTH); (**D**) indirect confrontation from the *F. oxysporum* side (vFO); and (**E**) indirect confrontation from the *T. harzianum* side (vTH). Compared with the monoculture control, *F. oxysporum* growth was significantly inhibited under both direct and indirect confrontation conditions, demonstrating the antagonistic activity of *T. harzianum* against the pathogen.

To examine how different confrontation modes influence antagonistic outcomes, *F. oxysporum* and *T. harzianum* were cultured under monoculture, direct confrontation, and indirect confrontation conditions. Compared with the monoculture control (cFO), direct confrontation significantly decreased the biomass of *F. oxysporum* (*p* = 0.028), while indirect confrontation also exhibited a significant inhibitory effect (*p* = 0.039) (Table S5, Figure 1). Notably, in the direct confrontation system, red pigment production in *F. oxysporum* was observed even prior to physical contact, and fungal growth was completely inhibited after hyphal interaction. In contrast, indirect confrontation resulted in widespread pigment accumulation across the entire colony after 7 days, whereas pigment production in monoculture conditions remained largely restricted to peripheral regions. These phenotypic variations indicate that physical contact induces an acute stress response, whereas indirect interactions promote a sustained chemically mediated defense state, possibly associated with polyketide-derived pigments.

*T. harzianum* displayed distinct growth patterns depending on the confrontation mode. Under direct confrontation, its overall biomass was slightly reduced compared with monoculture; however, hyphae in the interaction zone exhibited pronounced morphological changes and aggressively overgrew the pathogen colony (Table S6, Figure 1). This behavior is linked to contact-dependent mycoparasitism, which involves localized secretion of cell wall-degrading enzymes and antimicrobial compounds. In indirect confrontation, biomass reduction was less evident, and hyphal morphology was similar to that observed in monoculture, suggesting that diffusible signals exert relatively limited effects on *T. harzianum* growth. Overall, these findings demonstrate that direct and indirect confrontation impose different physiological stresses on both fungi, resulting in divergent growth behaviors and defense strategies.

### 3.2. Antagonism Induces Mode-Specific Metabolic Reprogramming in Both Fungi

**Figure 2:**
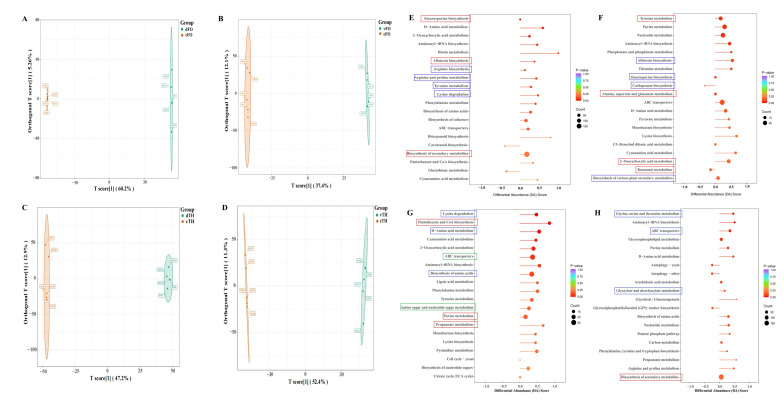
OPLS-DA analysis of metabolomic profiles in *F. oxysporum* and *T. harzianum* under confrontation conditions. (**A**,**C**) Direct confrontation versus monoculture; (**B**,**D**) indirect confrontation versus monoculture. (**E**) KEGG pathway enrichment analysis of differentially regulated metabolites in *F. oxysporum* under direct confrontation compared with monoculture; (**F**) KEGG analysis of *F. oxysporum* under indirect confrontation; (**G**) KEGG analysis of *T. harzianum* under direct confrontation; and (**H**) KEGG analysis of *T. harzianum* under indirect confrontation. Special Description: (**E**) Secondary metabolism-related pathways (red box) and amino acid biosynthesis and degradation pathways (blue box); (**F**) pathways associated with energy supply and organic acid metabolism (blue box) and secondary metabolism pathways of *F. oxysporum* (red box); (**G**) amino acid biosynthesis and degradation pathways (blue box), coenzyme synthesis and organic acid metabolism pathways (red box), as well as ABC transporters and amino sugar and nucleotide sugar metabolism pathways (green box); (**H**) selected pathways involved in protein synthesis, transmembrane transport, and amino acid utilization (blue box) and secondary metabolite biosynthesis pathways (red box). [1]: The first orthogonal component extracted.

Non-targeted metabolomics analysis revealed substantial metabolic differences between confrontation treatments and monoculture controls. Orthogonal partial least squares discriminant analysis (OPLS-DA) demonstrated clear separation of metabolic profiles for both fungi under direct and indirect confrontation conditions (Figure 2A–D). In *F. oxysporum*, samples from direct confrontation showed higher intra-group consistency, as indicated by lower orthogonal variance (5.26%), whereas indirect confrontation samples exhibited greater variability (12.1%), suggesting enhanced metabolic plasticity under non-contact stress.

Pathway enrichment analysis further revealed mode-specific metabolic alterations. In *F. oxysporum*, direct confrontation was linked to the upregulation of pathways associated with polyketide-derived secondary metabolites, including those annotated as aflatoxin-related biosynthetic pathways (Figure 2E). In contrast, indirect confrontation predominantly activated amino acid metabolism and energy-associated pathways, indicating maintenance of cellular homeostasis through resource redistribution under prolonged competitive stress (Figure 2F). In *T. harzianum*, both confrontation modes enhanced pathways related to ABC transporters, amino sugar and nucleotide sugar metabolism, and cell wall remodeling, reflecting conserved adaptive responses to antagonistic stress (Figure 2G,H).

**Figure 3:**
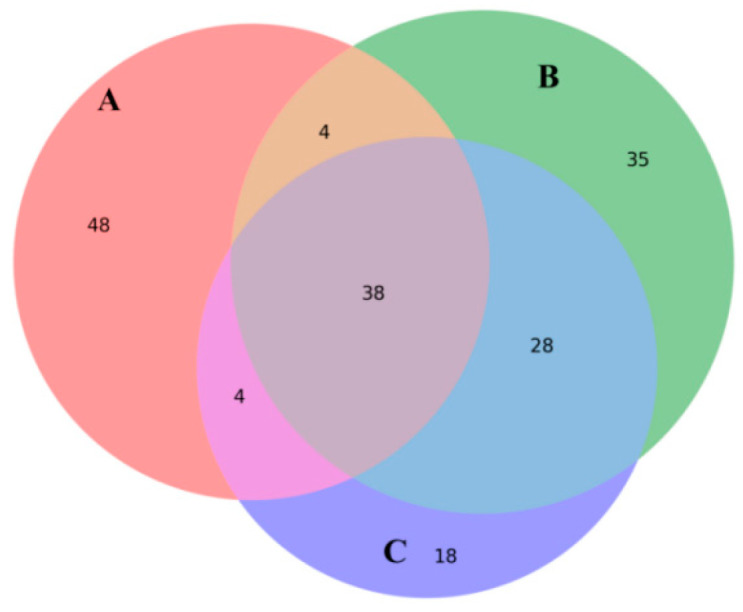
Venn diagram of mycotoxins. (**A**): *F. oxysporum* monoculture; (**B**): *F. oxysporum* in direct confrontation with *T. harzianum*; (**C**): *F. oxysporum* in indirect confrontation with *T. harzianum*.

**Figure 4:**
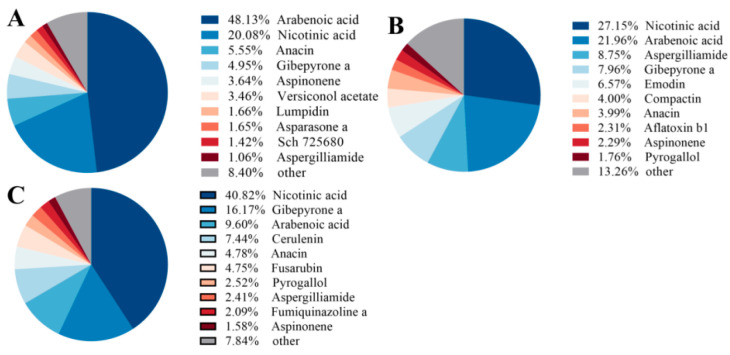
Relative abundance distribution of major metabolites. (**A**): *F. oxysporum* monoculture; (**B**): *F. oxysporum* in direct confrontation with *T. harzianum*; (**C**): *F. oxysporum* in indirect confrontation with *T. harzianum*. For each group, the top ten most abundant metabolites are shown with their relative contents; the remainder are grouped as “other”.

**Figure 5:**
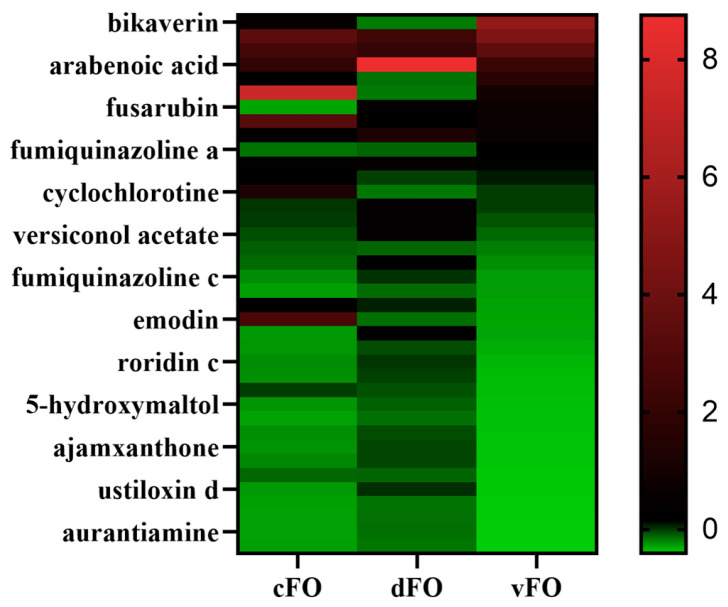
Heatmap of relative abundance of shared mycotoxins under different culture conditions. After Z-score normalization, relative expression levels of shared toxins in *F. oxysporum* monoculture (cFO), direct confrontation with *T. harzianum* (dFO), and indirect confrontation (vFO). Color gradient ranges from green (low expression) to red (high expression).

Targeted mycotoxin analysis identified 38 core mycotoxins consistently detected across all culture conditions. Monoculture samples contained the highest number of unique toxins, whereas direct confrontation produced 35 unique compounds and indirect confrontation yielded 18 (Figure 3). Direct confrontation preferentially enriched several virulence-associated metabolites, including aflatoxin B1 (AFB1) and emodin. In contrast, indirect confrontation was characterized by a higher relative abundance of nicotinic acid and a reduced proportion of several highly toxic metabolites among the top ten compounds (Figure 4). Heatmap analysis further revealed mode-dependent regulation of shared toxins: compounds such as aloe-emodin and rusticin C were strongly induced during confrontation, particularly under direct conditions, whereas others (e.g., patulin derivatives and dihydroxyisoflavones) exhibited selective sensitivity to specific interaction modes (Figure 5). These results indicate that the confrontation mode not only influences toxin levels but also determines the qualitative composition of the mycotoxin profile.

### 3.3. T. harzianum Exhibits Strong Concentration-Dependent Detoxification of Representative Mycotoxins

Based on the enrichment of aflatoxin biosynthesis pathways and the elevated AFB1 levels observed during direct confrontation, AFB1 was selected as a representative toxin for detoxification analysis. ZEN, a polyketide-derived mycotoxin sharing common biosynthetic precursors with dominant metabolites, and the exogenous T-2 toxin were included as additional representatives.

**Figure 6:**
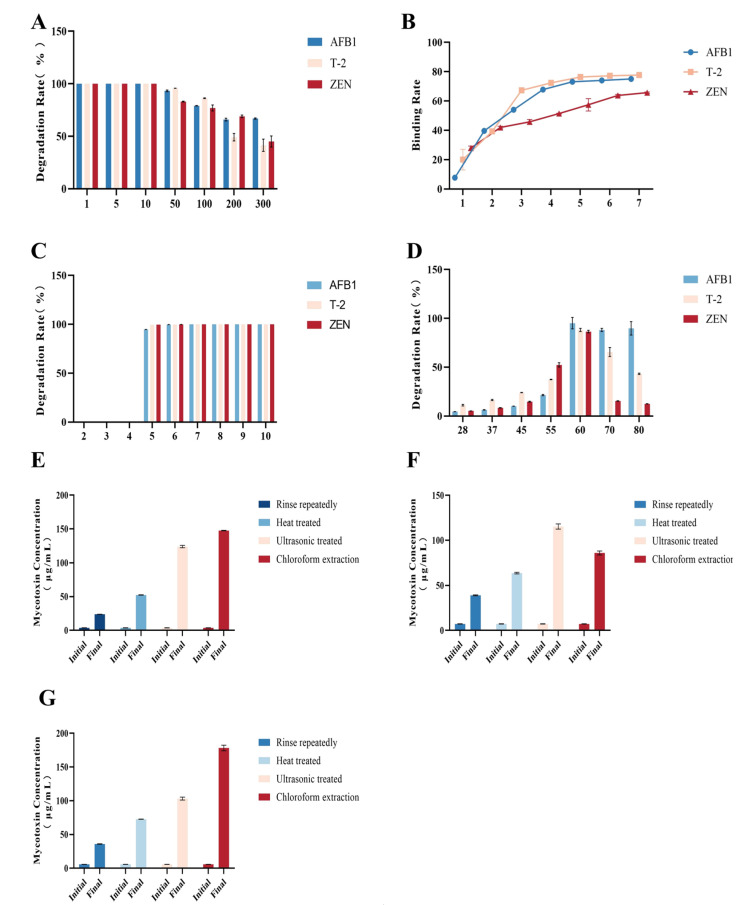
Detoxification effects of *T. harzianum* on representative mycotoxins. (**A**) Degradation efficiencies of three mycotoxins by *T. harzianum* at different initial concentrations. (**B**) Time-course binding rates of *T. harzianum* to AFB1, T-2 toxin, and ZEN. (**C**,**D**) Effects of different pH (**C**) and temperature (**D**) conditions on toxin degradation rates. (**E**–**G**) Residual toxin concentrations in *T. harzianum*–mycotoxin complexes following different treatments: (**E**) AFB1; (**F**) T-2 toxin; and (**G**) ZEN. Treatments included repeated rinsing, heat treatment, ultrasonic treatment, and chloroform extraction. Toxin concentrations were recorded before (Initial) and after (Final) each treatment.

Concentration-dependent experiments indicated that at low concentrations (1–10 μg/mL), degradation of all three toxins was nearly complete (>95%), suggesting that the detoxification system remained unsaturated under these conditions (Figure 6A). At concentrations ≥50 μg/mL, degradation efficiency progressively declined, consistent with saturation kinetics. AFB1 maintained relatively high degradation efficiency even at ≥100 μg/mL, whereas T-2 toxin reached a plateau at 200–300 μg/mL, and ZEN exhibited a sharp decrease at 100–200 μg/mL; these ranges were subsequently defined as saturation concentrations for downstream analyses.

Time-course analysis revealed distinct temporal binding patterns (Figure 6B). AFB1 and T-2 toxin rapidly associated with *T. harzianum* hyphae, reaching plateau levels at about 3 h and 4 h, respectively, whereas ZEN displayed slower kinetics, stabilizing at 6 h. Environmental factor experiments showed that detoxification efficiency remained high and stable across a wide pH range (5–10), with only a slight reduction for AFB1 at pH 5 (Figure 6C). Temperature analysis demonstrated a bell-shaped response, with maximum activity observed at 55–60 °C (Figure 6D). At temperatures above 70 °C, detoxification efficiency decreased markedly, particularly for ZEN, indicating partial thermal sensitivity of the underlying mechanism.

Stability experiments further clarified the nature of toxin-fungus interactions. Repeated washing released minimal toxin, indicating stable binding, whereas heat and ultrasonic treatments partially released bound toxins, especially T-2 and ZEN (Figure 6E–G). In contrast, chloroform extraction almost completely recovered all three toxins, suggesting that lipophilicity and lipid-associated interactions play a key role in toxin binding. Overall, these findings indicate that toxin reduction may involve reversible physical adsorption and possibly additional biochemical processes. However, no specific detoxification enzymes were identified, and degradation products were not structurally characterized in this study. Therefore, the underlying mechanisms remain unresolved.

### 3.4. Transcriptomics Analysis Reveals Core Regulatory Hubs Associated with Antagonistic Stress

**Figure 7:**
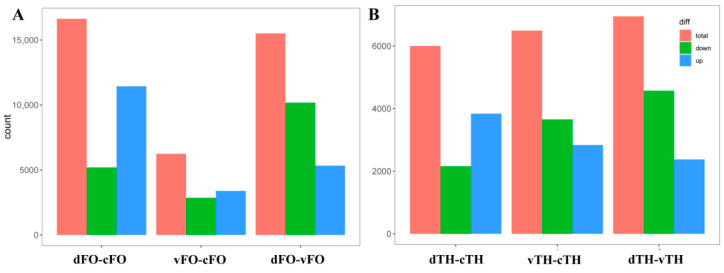
Bar chart showing the number of differentially expressed genes under different experimental conditions. cFO: *F. oxysporum* monoculture; dFO: *F. oxysporum* in direct confrontation with *T. harzianum*; vFO: *F. oxysporum* in indirect confrontation with *T. harzianum*; cTH: *T. harzianum* monoculture; dTH: *T. harzianum* in direct confrontation with *F. oxysporum*; vTH: *T. harzianum* in indirect confrontation with *F. oxysporum* (same abbreviations apply below). (**A**) Distribution of differentially expressed genes of *F. oxysporum* under different confrontation treatment conditions. (**B**) Distribution of differentially expressed genes of *T. harzianum* under different confrontation treatment conditions.

**Figure 8:**
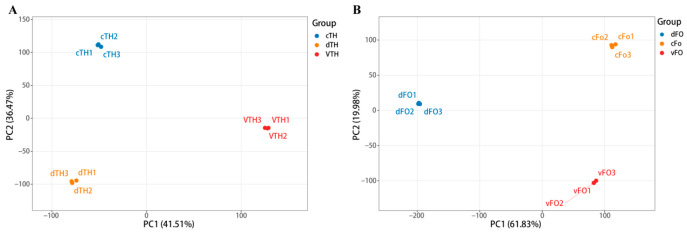
PCA score plot of differentially expressed genes. (**A**) *T. harzianum* PC1 and PC2 account for 41.51% and 36.47% of the total variance, respectively. Control, direct confrontation, and indirect confrontation samples form distinct clusters with clear intergroup separation and good intragroup reproducibility. (**B**) *F. oxysporum* PC1 and PC2 account for 61.83% and 19.98% of the total variance, respectively. Samples are tightly grouped by treatment, with an obvious separation trend among the different groups.

RNA sequencing identified more than 2000 differentially expressed genes (DEGs) in each fungus under confrontation conditions relative to monoculture (Figure 7 and Figure S3). Principal component analysis (PCA) further confirmed distinct transcriptional states corresponding to the different confrontation modes (Figure 8).

In *F. oxysporum*, *DEGs* were significantly enriched in glutathione metabolism, coenzyme Q and other terpenoid-quinone biosynthesis, and polyamine metabolism (Figures S4 and S5). Ten candidate pathogenicity-related genes, including *Coq6*, *ODC*, and *NAT*, were selected for further analysis (Table S7). Integrated transcriptomic-metabolomic analysis revealed coordinated regulation of coenzyme Q biosynthesis and glutathione metabolism, highlighting their central roles in redox balance and stress adaptation during antagonistic interactions (Figures S6 and S7).

**Figure 9:**
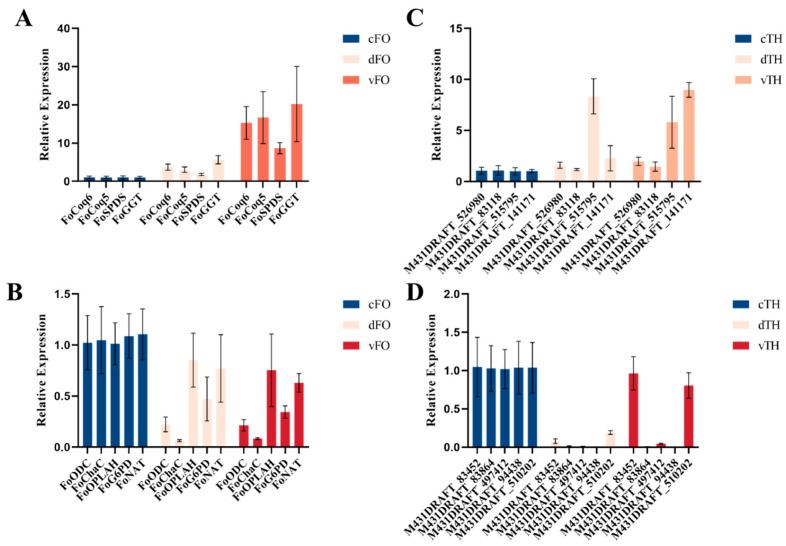
Relative expression levels of target genes in *F. oxysporum.* (**A**,**B**) and hypothetical protein genes in *T. harzianum* (**C**,**D**). (**A**,**C**): Upregulated genes (*Coq6*, *Coq5*, *SPDS*, *GGT*); (**B**,**D**): Downregulated genes (*ODC*, *ChaC*, *OPLAH*, *G6PD*, *NAT*).

In *T. harzianum*, *DEGs* were mainly associated with cell wall remodeling, polysaccharide metabolism, glycolysis/gluconeogenesis, and steroid biosynthesis (Figures S8–S11). Several strongly induced hypothetical proteins contained conserved domains related to redox regulation and molecular transport (Table S8), indicating previously uncharacterized roles in mycoparasitism and stress tolerance. RT-qPCR validation confirmed the expression patterns of representative genes in both fungi (Figure 9).

### 3.5. dsRNA-Mediated Gene Silencing Validates Pathogenicity Targets and Supports Scalable RNAi Applications

**Figure 10:**
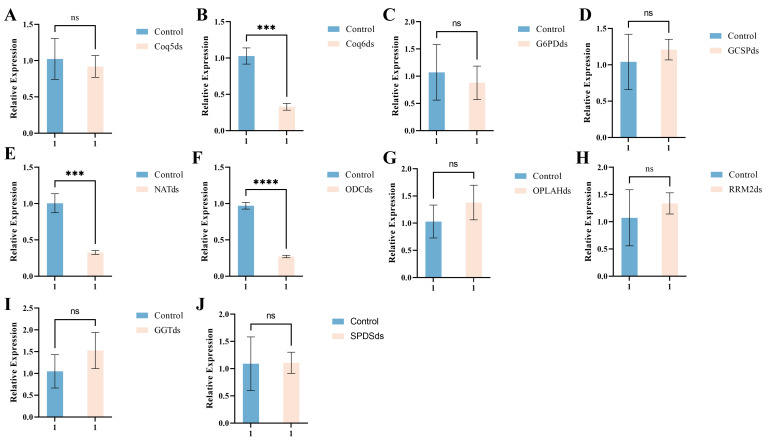
Effects of dsRNA treatment on target gene expression in *F. oxysporum*. ((**A**): *Coq5*, (**B**): *Coq6*, (**C**): *G6PD*, (**D**): *GCSP*, (**E**): *NAT*, (**F**): *ODC*, (**G**): *OPLAH*, (**H**): *RRM2*, (**I**): *GGT*, (**J**): *SPDS*) Relative expression levels of the 10 target genes before and after dsRNA treatment. Bars represent mean ± SEM (*n* = 3 biological replicates). Statistical differences were evaluated by two-sided Student’s *t*-test, with significance indicated as: ns (no significant difference), *** *p* < 0.001, **** *p* < 0.0001.

To functionally validate candidate pathogenicity-related genes, dsRNAs targeting ten genes in *F. oxysporum* were synthesized and verified by agarose gel electrophoresis (Figure S12). dsRNA treatment significantly reduced the transcript levels of several target genes, including *Coq6* (*p* = 0.0008), *NAT* (*p* = 0.0007), and *ODC* (*p* < 0.0001). In contrast, no significant differences were detected in the expression of several other genes. However, no phenotypic assays (e.g., growth inhibition or virulence reduction) were performed in this study, and therefore the functional consequences of gene silencing remain to be validated (Figure 10).

**Figure 11:**
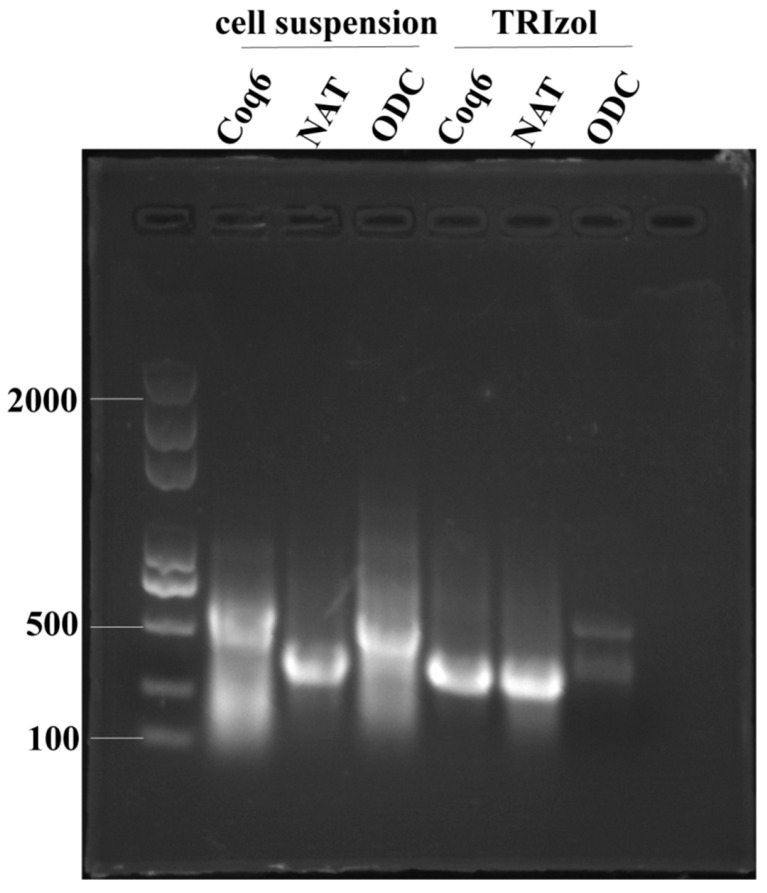
Agarose gel electrophoresis analysis of dsRNA extracted by the ethanol fixation method (cell suspension) and TRIzol method from the HT115(DE3)/L4440 expression system.

**Figure 12:**
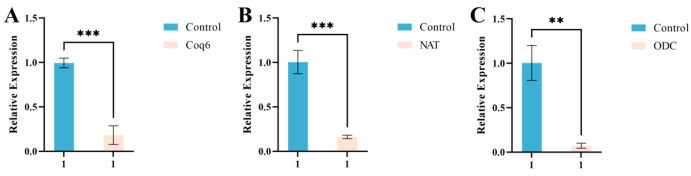
RT-qPCR results showing the effects of dsRNA extracted by the ethanol fixation method from the HT115(DE3)/L4440 expression system on target genes in *F. oxysporum*. (**A**–**C**) Treatment with *Coq6* dsRNA, *NAT* dsRNA, and *ODC* dsRNA significantly reduced the expression of the respective target genes. These results demonstrate that dsRNA prepared by the 75% ethanol fixation method retains effective silencing activity and can reliably induce target gene knockdown in *F. oxysporum*. ** *p* < 0.01, *** *p* < 0.001.

To facilitate scalable dsRNA production, selected dsRNAs were expressed in the HT115(DE3)/L4440 *E. coli* system and extracted using either the 75% ethanol fixation method or the conventional TRIzol method. Both methods produced dsRNA with comparable purity and concentration, with A260/A280 ratios exceeding 2.1 and no significant variation in yield (Table S9, Figure 11). Importantly, dsRNA obtained via ethanol fixation retained strong silencing activity, as indicated by significant suppression of target gene expression in *F. oxysporum* at a final concentration of 50 μg/mL (Figure 12). These findings demonstrate that dsRNA-mediated RNAi effectively targets key pathogenicity genes, while the ethanol-fixed *E. coli* system provides a cost-effective and stable platform for large-scale dsRNA production.

## 4. Discussion

The antagonistic interaction between *T. harzianum* and *F. oxysporum* represents a complex and dynamically regulated process involving physical contact, chemical communication, and molecular responses. These mechanisms differ substantially depending on the interaction mode. Based on integrated multi-omics analyses, this study comparatively characterizes that direct confrontation is primarily associated with contact-dependent stress responses and mycoparasitism, whereas indirect confrontation is more closely related to metabolic reprogramming and signal-mediated adaptive regulation.

During direct confrontation, *T. harzianum* strongly suppresses *F. oxysporum* through hyphal coiling, cell wall degradation, and secretion of antifungal metabolites. This observation is consistent with classical mycoparasitic mechanisms [29,30]. Notably, *F. oxysporum* accumulated red pigments prior to physical contact, which may be linked to activation of polyketide pathways and could represent a preemptive response to oxidative stress [31]. In contrast, indirect confrontation resulted in weaker inhibition but induced broader metabolic changes. This suggests that diffusible signals play a significant regulatory role in fungal interactions.

Metabolomic analysis revealed distinct responses under the two confrontation modes. In direct confrontation, several polyketide-related pathways in *F. oxysporum*, including those annotated as aflatoxin-like biosynthesis, were enriched. This may indicate a stress response characterized by enhanced secondary metabolism [32]. In contrast, indirect confrontation redirected metabolic flux toward amino acid cycling and energy metabolism, which may help sustain cellular homeostasis under competitive conditions. It should be noted that metabolite identification was largely based on database annotation. Therefore, these pathway-level changes should be interpreted as indicative trends rather than definitive evidence of specific metabolic pathways.

Regarding toxin-related metabolism, 38 core mycotoxin-associated metabolites were identified. Under direct confrontation, several bioactive compounds, including anthraquinones, exhibited increased abundance, which is consistent with reports of stress-induced toxin production in *Fusarium* species [33]. In contrast, indirect confrontation did not increase toxin diversity but instead altered metabolic composition and reduced the abundance of some highly toxic metabolites. These observations suggest that different interaction modes influence toxin metabolism through resource allocation; however, their direct relationship with pathogenicity remains to be experimentally validated.

The detoxification capability of *T. harzianum* provides an additional antagonistic mechanism. In this study, toxin degradation exhibited concentration dependence and reached optimal activity at pH 6–7 and 55–60 °C, with partial resistance to washing [34,35]. These effects may arise from a combination of surface adsorption and potential biochemical processes. However, no specific detoxification enzymes were identified, and the degradation products were not structurally characterized in this study. Therefore, the underlying mechanisms remain unresolved and should be interpreted with caution. Future studies employing targeted analytical techniques (e.g., LC–MS/MS-based structural identification and enzyme isolation) will be necessary to clarify these processes.

Integrated transcriptomic and metabolomic analyses showed that glutathione metabolism and ubiquinone biosynthesis were enriched in *F. oxysporum*, likely reflecting adaptive responses to ROS stress [36,37]. Polyamine metabolism may also contribute to stress tolerance and regulation of virulence [38]. These results are consistent with previous studies highlighting the role of antioxidant systems in pathogen adaptation. For example, Batool et al. (2026) demonstrated that modulation of antioxidant enzyme systems can significantly alleviate *Fusarium*-induced stress, supporting the idea that redox regulation is a central component of fungal stress responses [39]. Similarly, beneficial microorganisms have been shown to suppress *Fusarium* diseases by activating induced systemic resistance (ISR) and systemic acquired resistance (SAR) in plants, indicating that stress-response pathways are closely linked to disease suppression mechanisms [40]. Taken together, these findings suggest that the redox and metabolic responses observed in this study may represent conserved adaptive strategies during antagonistic interactions, although their direct contribution to pathogenicity remains to be further validated.

RNAi experiments further supported the functional importance of several key genes. To improve dsRNA specificity, target fragments were designed based on gene-specific regions and evaluated by genome-wide alignment to minimize off-target effects [41]. However, the current RNAi results are limited to transcriptional knockdown, and no phenotypic validation (e.g., growth inhibition or virulence assays) was performed. Therefore, the functional significance of these genes in pathogenicity remains uncertain. In addition, although off-target effects were computationally minimized, they cannot be completely excluded, and the absence of non-specific dsRNA controls represents a limitation of this study. Future studies should incorporate non-specific controls, multi-target strategies, and transcriptome-wide analyses to further validate RNAi specificity and assess long-term effects.

Despite the comprehensive multi-omics approach, several limitations should be considered. First, the experiments were conducted in vitro and may not fully represent plant–soil environments [42]. Second, metabolite identification relied on database annotation, which may introduce potential misannotation. Third, RNAi experiments mainly evaluated transcriptional responses, whereas long-term phenotypic effects remain unclear. In addition, dsRNA delivery efficiency and environmental stability remain major challenges for practical application [43].

Overall, this study highlights the dynamic and mode-dependent nature of fungal antagonistic interactions. Direct contact induces strong stress responses and enhanced secondary metabolism, whereas indirect interaction promotes metabolic adjustment and signal-mediated regulation. Importantly, this work provides a comparative and systems-level perspective rather than definitive mechanistic conclusions. Although combining biological control with RNAi shows potential, this integration is presented here as a conceptual framework and was not experimentally validated in this study. Future research should evaluate this strategy under plant-associated and field conditions to determine its practical feasibility. In addition, emerging eco-friendly approaches, such as green-synthesized silver nanobiofungicides, have been reported to effectively control Fusarium-related diseases [44], highlighting the potential of integrating multiple strategies for sustainable disease management. However, the compatibility and effectiveness of combining such approaches with microbial biocontrol or RNAi-based strategies require further investigation.

## Data Availability

The original contributions presented in this study are included in the article/supplementary material. Further inquiries can be directed to the corresponding author.

## Supplementary Materials

The following supporting information can be downloaded at: https://www.mdpi.com/article/10.3390/microorganisms14051068/s1, Table S1: Detailed LC gradient elution programs for LC–MS analyses; Figure S1: Liquid chromatography standard calibration curves of mycotoxins; Table S2: List of primers used in this study; Table S3: dsRNA synthesis regions of antagonistic target genes screened in *F. oxysporum*; Table S4: Sequence characteristics of each dsRNA; Figure S2: Construction of L4440 plasmids carrying dsRNA fragments targeting *F. oxysporum *genes; Table S5: Biomass and inhibition rate of *F. oxysporum* under different confrontation conditions; Table S6: Biomass and inhibition rate of *T. harzianum *under different confrontation conditions; Figure S3: Heatmap showing the relative abundance of differential metabolites under different treatments; Figure S4: Directed acyclic graph of enriched Gene Ontology biological processes in *F. oxysporum* under direct confrontation with *T. harzianum*; Figure S5: Directed acyclic graph of enriched Gene Ontology biological processes in *F. oxysporum* under indirect confrontation with *T. harzianum*; Table S7: Key differentially expressed genes of *F. oxysporum* under interspecific confrontation conditions; Figure S6: Ubiquinone and other terpenoid-quinone biosynthesis pathway; Figure S7: Glutathione metabolism pathway; Figure S8: Directed acyclic graph of enriched Gene Ontology biological processes in *T. harzianum* under direct confrontation with *F. oxysporum*; Figure S9: Directed acyclic graph of enriched Gene Ontology biological processes in *T. harzianum* under indirect confrontation with *F. oxysporum*; Figure S10: Glycolysis/Gluconeogenesis pathway; Figure S11: Steroid biosynthesis pathway; Figure S12: Agarose gel electrophoresis of chemically synthesized dsRNAs targeting *F. oxysporum* genes; Table S8: Putative hypothetical protein genes screened in *T. harzianum* and their conserved domains; Table S9: A260 and A280 absorbance values and concentrations of dsRNA obtained using different extraction methods.
