# Supplementary material for: Molecular Mechanisms and Metabolic Responses in the Biological Antagonism Between Trichoderma harzianum and Fusarium oxysporum
Source: Microorganisms. 2026 May 9;14(5):1068. doi: 10.3390/microorganisms14051068 (PMC13210364; doi:10.3390/microorganisms14051068)
Supplement: Supplementary file 1 [file microorganisms-14-01068-s001.zip › microorganisms-4207312-supplementary.pdf]

Supplementary Materials

Table S1. Detailed LC gradient elution programs for LC–MS analyses.

Non-targeted metabolomics analysis

Time (min)	Mobile phase B (%)	Flow rate (mL·min^{-1})
0.0	5	0.4
1.0	5	0.4
9.0	95	0.4
12.0	95	0.4
12.1	5	0.4
15.0	5	0.4

Mobile phase A: water with 0.1% formic acid, Mobile phase B: acetonitrile with 0.1% formic acid, Column: Waters ACQUITY Premier HSS T3, Injection volume: 4 μL, Ionization mode: ESI$^+$/ESI$^-$.

Targeted mycotoxin analysis

Time (min)	Mobile phase B (%)	Flow rate (mL·min^{-1})
0.0	20	1.0
0.1	20	1.0
10.0	90	1.0
15.0	90	1.0
20.0	20	1.0

Mobile phase A: water with 0.1% formic acid, Mobile phase B: acetonitrile with 0.1% formic acid, Column: C18 analytical column (4.6 mm × 250 mm), Injection volume: 10 μL, Ionization mode: ESI$^+$/ESI$^-$.

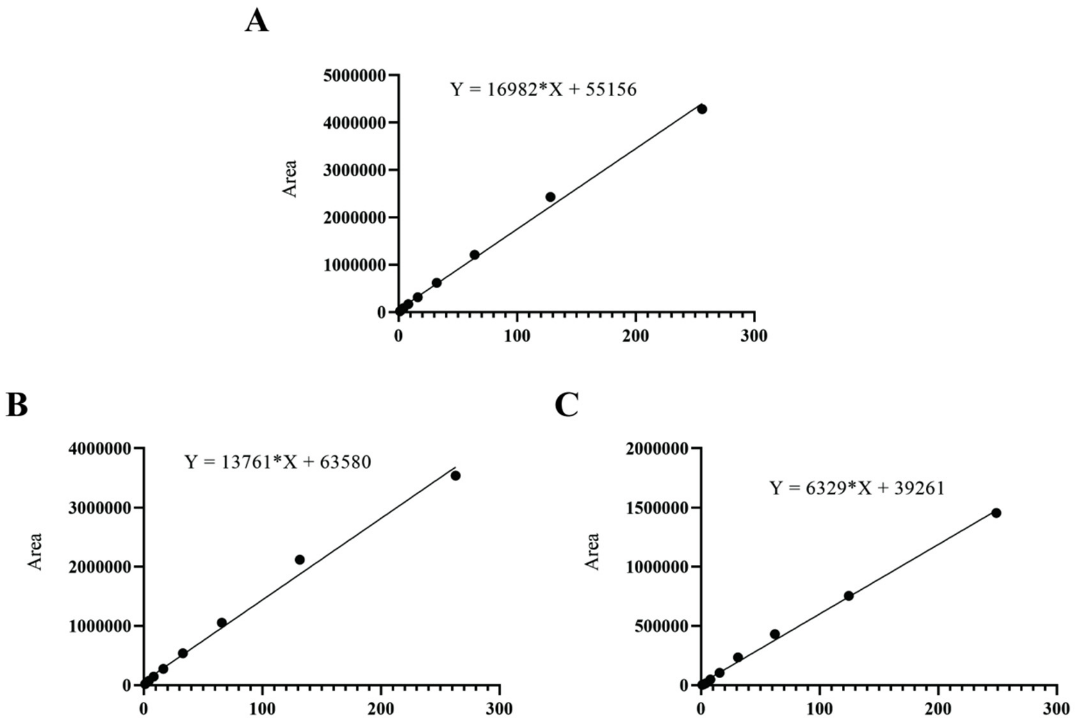

Figure S1. Liquid chromatography standard calibration curves of mycotoxins.
(A) Aflatoxin B1; (B) T-2 toxin; (C) zearalenone. The x-axis represents the concentration of each target mycotoxin (μg/mL), and the y-axis represents the corresponding peak area.

Table S2. List of primers used in this study.

Primer name	Sequence (5′–3′)
Focoq2dsF	taatacgactcactatagggTTGCGACCCTTAGCAAGAGG
Focoq2dsR	taatacgactcactatagggGTTGCTCCCCCACAGCTAAT
Focoq6dsF	taatacgactcactatagggGCTTCCACTTCGACACAGGA
Focoq6dsR	taatacgactcactatagggACTGGCCATTCGCTCAGATC
FocoqdsF	taatacgactcactatagggCCTACTTTCGTCCGGCCATT
Focoq5dsR	taatacgactcactatagggGGCAACTGTGTAAAGATCGAGC
FoSPDSdsF	taatacgactcactatagggACATCGACGAGGCTGTTGTC
FoSPDSdsR	taatacgactcactatagggTTCTGCCGTTCCTAGGGACG
FoODCdsF	taatacgactcactatagggGCTGTCCTCGACACCTACAA
FoODCdsR	taatacgactcactatagggCTCCTGGATAGAGCTTGGCG
FoRRM2dsF	taatacgactcactatagggGTGCCAGCAAGCAGATGATC
FoRRM2dsR	taatacgactcactatagggACTCGTCCTCCTCAGTCTTCT
FoGCSPdsF	taatacgactcactatagggCGTGTTGCTGAGGACAAGGT
FoGCSPdsR	taatacgactcactatagggTGATACAGCGAAGACCAGCA
FoGGTdsF	taatacgactcactatagggTCAACAACGACACCCTCAAGT
FoGGTdsR	taatacgactcactatagggTCTGAACCTGGTACTCACCCA
FoOPLAHdsF	taatacgactcactatagggTTGGGCTTCGATATGGGTGG
FoOPLAHdsR	taatacgactcactatagggTGAAGACAGCGTGTTGACCA
FoG6PDdsF	taatacgactcactatagggCGAGTTCGGTATCGTTCGTG
FoG6PDdsR	taatacgactcactatagggGGTGAGATCCAGCTCGGTAA
FoNATdsF	taatacgactcactatagggCGACTGGTCCATCTCATCGAG
FoNATdsR	taatacgactcactatagggGTCCAAATTGGGTGTTGTCCA
FoTTKdsF	taatacgactcactatagggGATCTCGACCTCACAGCCGA
FoTTKdsR	taatacgactcactatagggATCCTCCCCGGGTTGACTCT
FoCoq2F	AGCATGGTGGGACTGTTAGC
FoCoq6F	CTGGCCAGACAAACCCTCAA
FoCoq5F	AGTTGCCTTCGGTATTCGCA
FoSPDSF	ATTTTGGTGCCGCTTCCCTC
FoODCF	CTTCGAATCAGCAGAGCCCA
FoCoq2R	GATCATAGCACCGAGCGTCA
FoCoq6R	TGCTCGAGCGAGAAGCTATG
FoCoq5R	TGGGGTGCTTGTCAACCTTT
FoSPDSR	GCGGATCAGGAACAGCAGAA
FoODCF	AGGAGAGAAAGTGGATGGGGA
FoGGTF	TGACGCGGAAGAGATATGCC
FoChaCF	CCACACTTCGACCGCAGAAT
FoOPLAHF	ACTCCAGTATCCTGTCCGCT
FoG6PDF	TTCTCGTGGTCCTGCTGTTC

Primer name	Sequence (5′–3′)
FoNATF	GCCAAGAACCAGGCACAAAG
FoGGTR	ATCCGTCGTCATGATACCGC
FoChaCR	AAGGCATACCTAGCCTGCCA
FoOPLAHR	AGAGAGACTCAAGACGGGCT
FoG6PDR	AGGCGTTATTAGAGGATGCAAGT
FoNATR	GAGGCGTGAGTCCGTAGATG
ThD24SRF	ACGGAGGTTTCAGAGAACGG
6315F	CCTGCACAAAAACTCGTCGG
526980F	TGTATTCCGATGCTGGACGG
83118F	GGAGATTCGCATCAAACGGC
83118F	GCCAGTGCACATTTCAAGGG
515795F	CTTCCAACAAGTCGGTTGCG
ThD24SRR	GATGAAGTCGTCGTGGCTCT
6315R	TGTCGAACGGCTTCAGTCTC
526980R	AAACAAGCCGACGCTGTCTA
83118R	TTGTCAGTAAACCGGCCCTC
83118R	CCTCGTTGACACCCATCACA
515795R	AAAGGGGAACTTCTGCTCCG
83452F	GCTCAACAGACAACACAGCG
497412F	ACCACCGTCACTCTTTCGAC
497412F	CACAAGTTCCTTGCTGGTGC
94438F	GCGAGCCCCTTTTCTCTTCT
510202F	CCCATTGGCACTTCCCTCTT
83452R	GCCAAGAAGCCCTTACCCAT
497412R	GACACCAGGGAAGGGAACAG
497412R	CAGACCACCAATGACGGGAA
94438R	GTACTTGGCGGTGGTCTCAA

Table S3. dsRNA synthesis regions of antagonistic target genes screened in *F. oxysporum.*

Sequence	Start position	End position	GC content	Repeats	Palindromic sequences	Hairpin structures	Predicted targets
FoCoq2dsRNA	502	1016	46%	0	3	1	3
FoCoq6dsRNA	85	593	53%	0	0	1	3
FoCoq5dsRNA	61	576	53%	3	2	1	3
FoSPDSdsRNA	731	1036	56%	0	0	1	3
FoODCdsRNA	16	526	53%	1	1	1	3
FoRRM2dsRNA	350	661	54%	0	0	1	2
FoGCSPdsRNA	1495	2005	54%	2	1	1	3
FoGGTdsRNA	530	1060	51%	4	1	1	4
FoOPLAHdsRNA	964	1492	55%	0	2	1	4
FoG6PDdsRNA	537	1041	51%	1	4	1	4

Sequence	Start position	End position	GC content	Repeats	Palindromic sequences	Hairpin structures	Predicted targets
FoNATdsRNA	1489	1896	51%	0	2	1	4
FoTTKdsRNA	539	1047	54%	2	3	0	4
FoNdpkdsRNA	350	667	61%	0	2	1	3

Table S4. Sequence characteristics of each dsRNA.

dsRNA	Predicted feature	Sequence (5′–3′)
Focoq2dsRNA	Palindromic sequence	GGAATTCC
		TGATATCA
		CCATATGG
	Hairpin structure	CCTTAAAACACAAGG
	Predicted target	CCCACCTCATGTATACTATAT
		GGCGTTGATATCATATCAGAT
		AAAACACAAGGCGAATACGAA
Focoq6dsRNA	Hairpin structure	GAGTCGCCCTC
	Predicted target	AGGACAATATCTATGATGTTG
		AGGTTTGGGATGGAGTGACAG
		CTCAAGAGACTACAAGAACTC
	Repeat	ATCAACCCCGA
		GTTGCTGG
		CCGACATC
Focoq5dsRNA	Palindromic sequence	GCGATATCGC
		CGATATCG
	Hairpin structure	GAATGATTTC
	Predicted target	CGACATCTGATCGACCTACAC
		CGCCGAATCCTATGATAAGAT
		GGATAACTCGCTCGATCTTTA
FoSPDSdsRNA	Hairpin structure	GCTACTTCCAGC
	Predicted target	TGCGGTTTCGACCATCCCAAG
		TTCCTCAACGACTACAAGAAC
		TGTATGTTCCCTATTTTCTTG
	Repeat	CGTCAAGT
FoODCdsRNA	Palindromic sequence	GTGATCAC
	Hairpin structure	AGACACTTTCT
	Predicted target	CCTCGACACCTACAATCATAA
		GCGTGATCACTCCAAAGCAAC
		GTCAAACAGATGACTTTTGAC
FoRRM2dsRNA	Hairpin structure	AGAACATCT
	Predicted target	AGCAAGCAGATGATCCAGGAT
		CCCTTCGACTTCATGGAGAAC
	Repeat	CTCGGCGATCC
		GATGACCTA
		TCAAGTTC
		CAGAAGAG
FoGGTdsRNA	Palindromic sequence	TTCATGAA
	Hairpin structure	GATGCGACTATC
	Predicted target	CCTCAAGTTCATGAAGATGAC
		CTGCTGGAAGATTACTCAAAT
		TCCCAGAAGAGTCAACGATAG
		AGTTCGGTTACGGAATGCGAA

FoOPLAHdsRNA	Palindromic sequence	CAGCGCTG
		ATCGCGAT
	Hairpin structure	AGGCGGTCCCT
	Predicted target	CCAGTACAGATGTCGCAAGAT
		TGGGAGAACGGACTCCTCAAG
		CGATGTTGTGAGAGAAAAGTT
		TGCGACAATGACACGACCCAT
	Repeat	CAAGAACG
FoG6PDdsRNA	Palindromic sequence	GGATATCC
		ATTCGAAT
		ATGCGCAT
	Hairpin structure	GATTCGAATC
		TCCCCAAGGA
	Predicted target	AGCCCAAGAACGTCATCATTG
		AGCAGAAGACCGAGATTCGAA
		ACGAGCTTGTTATGCGCATTC
		ATATCAAGATGAACTCTAAG
FoNATdsRNA	Palindromic sequence	TATCTAGATA
		ATCTAGAT
	Hairpin structure	TGGACGACCA
	Predicted target	GGACTCATTTGGCAGAAAACT
		CCGAAGGCTTGGACGACCATA
		AGGAAGGATAATCCGGTGAAC
		CTGGACTATGTTCTGGACAAC
FoTTKdsRNA	Repeat	TCCAAGCA
		CGATGGAG
	Palindromic sequence	GACGCGTC
		TCGCGCGA
		TAATATTA
	Predicted target	CGCGATATGTCTTCTGACGAG
		TGCTAGATAGATCTGCTATTG
		ACGACCTGAGGATAATATTAA
		TGGAGAATTGATATTGTCTAG
FoNdpkdsRNA	Repeat	CAAGAACG
	Palindromic sequence	CAAGCTTG
		CATCGATG
	Hairpin structure	GACTCCGTC
	Predicted target	GGCAAGGAGCACCTCGAGAAG
		CCTTTCTTCCCTGGTCTGATT
		AGAACGCCCAGAAGGAGATTG

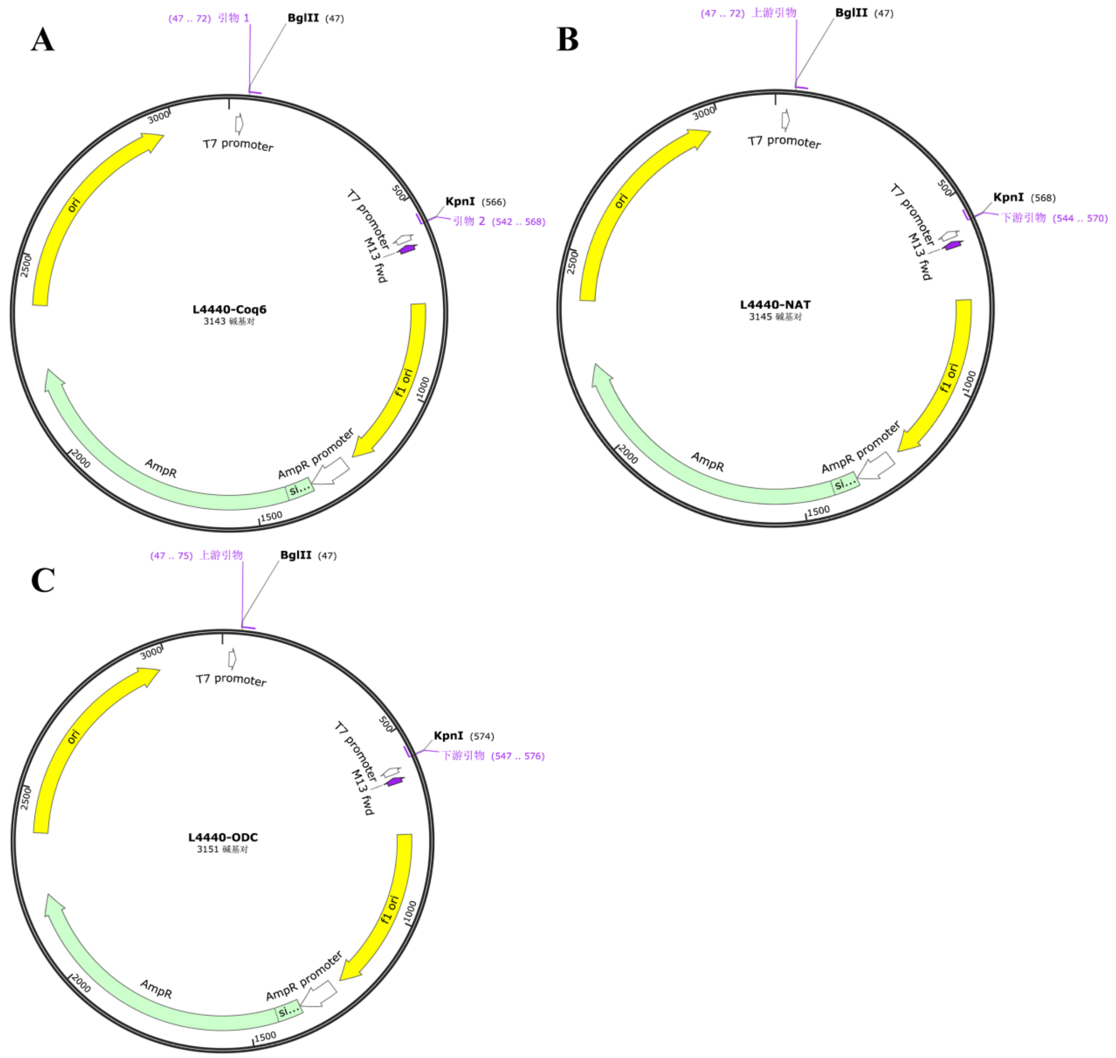

Figure S2. Construction of L4440 plasmids carrying dsRNA fragments targeting *F. oxysporum* genes.

(A) L4440-Coq6 plasmid containing a fragment of the *Coq6* gene; (B) L4440-NAT plasmid containing a fragment of the *NAT* gene; (C) L4440-ODC plasmid containing a fragment of the *ODC* gene.The restriction sites BglII and KpnI were used for cloning the upstream and downstream primers, respectively.

Table S5. Biomass and inhibition rate of *F. oxysporum* under different confrontation conditions.

Group	cFO	dFO	vFO
Biomass（mg）	0.420 ± 0.174a	0.083 ± 0.016c	0.113 ± 0.025b
Inhibition rate（%）	NA	78% ± 7.8%	68% ± 4.7%

Different lowercase letters indicate significant differences among groups based on one-way ANOVA followed by Tukey's multiple comparison test ($p < 0.05$).

Table S6. Biomass and inhibition rate of *T. harzianum* under different confrontation conditions.

Group	cTH	dTH	vTH
Biomass（mg）	0.462 ± 0.047a	0.169 ± 0.0753c	0.345 ± 0.083b

Inhibition rate（%）	NA	63% ± 12.5%	24.5% ± 5.7%

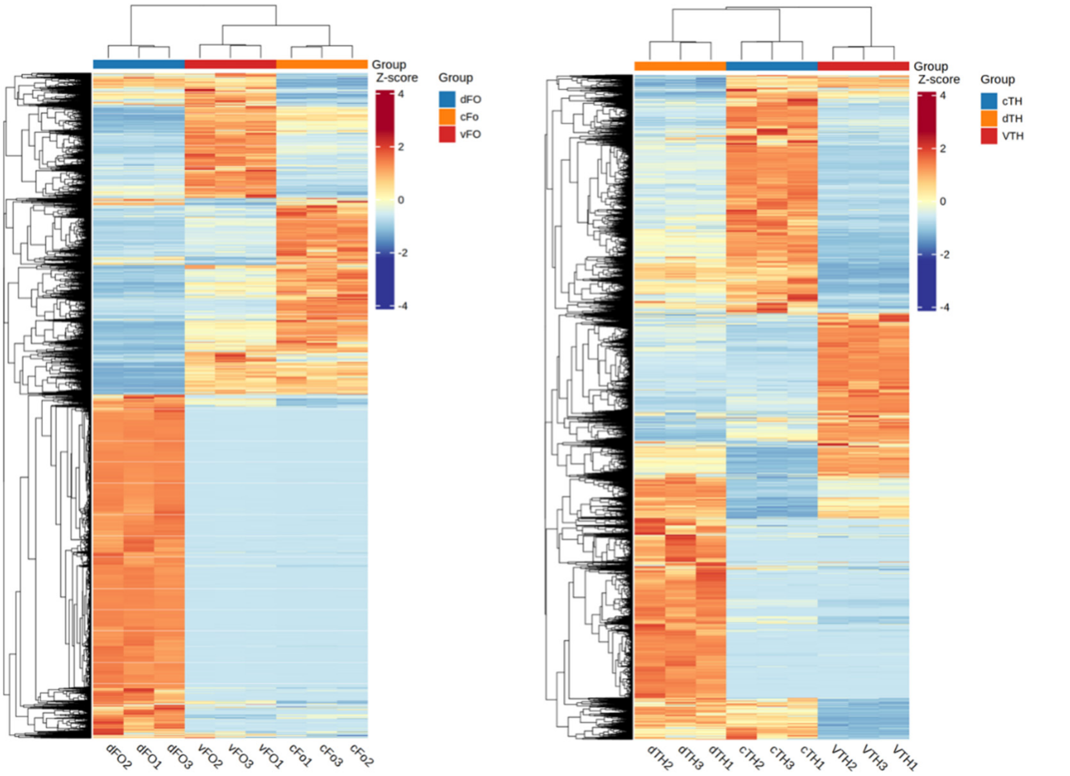

Figure S3. Heatmap showing the relative abundance of differential metabolites under different treatments.

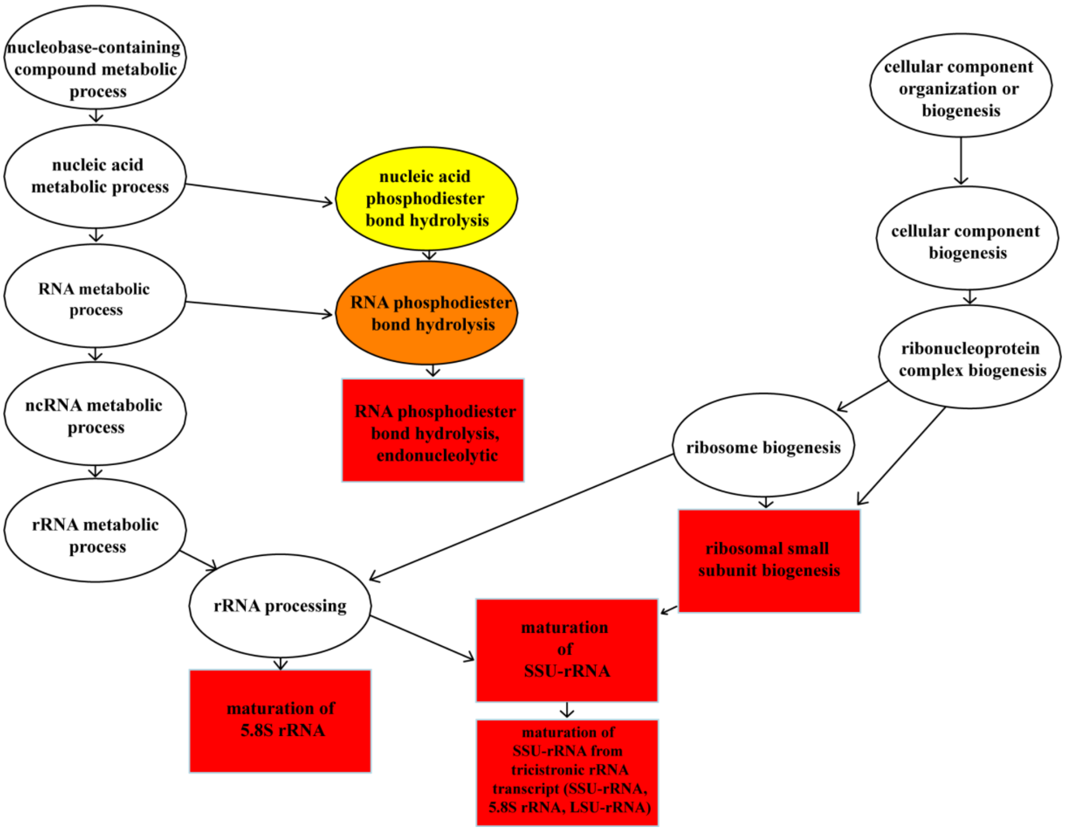

Figure S4. Directed acyclic graph of enriched Gene Ontology biological processes in *F. oxysporum* under direct confrontation with *T. harzianum*.

Each node represents a significantly enriched Gene Ontology biological process, and edges indicate hierarchical relationships among terms. Color intensity reflects the level of statistical significance, with white indicating no significant enrichment.

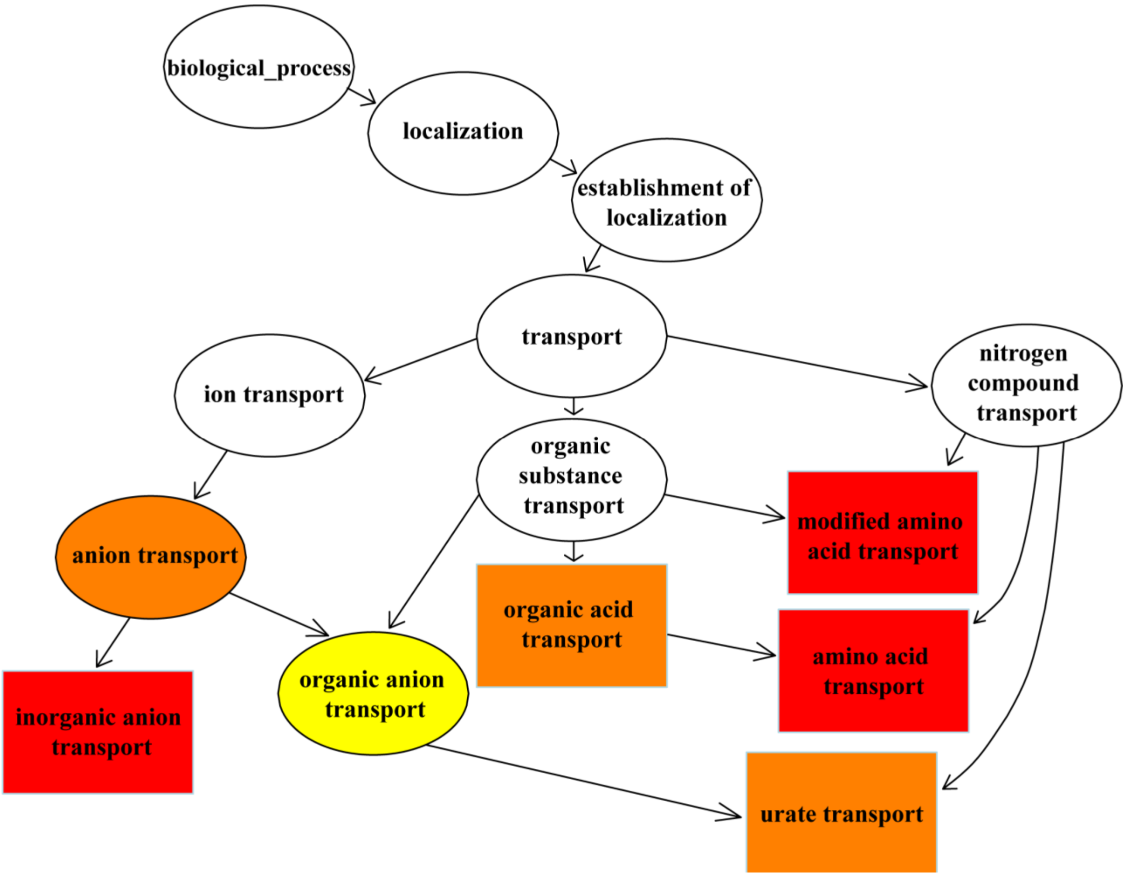

Figure S5. Directed acyclic graph of enriched Gene Ontology biological processes in *F. oxysporum* under indirect confrontation with *T. harzianum*.
Each node represents a significantly enriched Gene Ontology biological process, and edges indicate hierarchical relationships among terms. Color intensity reflects the level of statistical significance, with white indicating no significant enrichment.

Table S7. Key differentially expressed genes of *F. oxysporum* under interspecific confrontation conditions.

Gene ID	Gene name	Associated metabolic pathway
FOBCDRAFT_182054	*Coq2*(4-hydroxybenzoate polyprenyltransferase)	Ubiquinone biosynthesis
FOXG_00681	*Coq6*(ubiquinone biosynthesis monooxygenase)	Ubiquinone biosynthesis
FOBCDRAFT_31761	*UbiE/Coq5* (methyltransferase)	Ubiquinone biosynthesis
FOXG_18989	*SPDS* (spermidine synthase)	Polyamine metabolism
FOXG_07603	*ODC* (ornithine decarboxylase)	Polyamine metabolism
FOBCDRAFT_292483	*GGT* (γ-glutamyl transpeptidase)	Glutathione metabolism
FOIG_01132	*ChaC* (cation transport protein)	Amino acid metabolism
FOXG_05720	*OPLAH* (5-oxoprolinase)	Glutathione metabolism
FOXG_00106	*G6PD*(glucose-6-phosphate dehydrogenase)	Pentose phosphate pathway

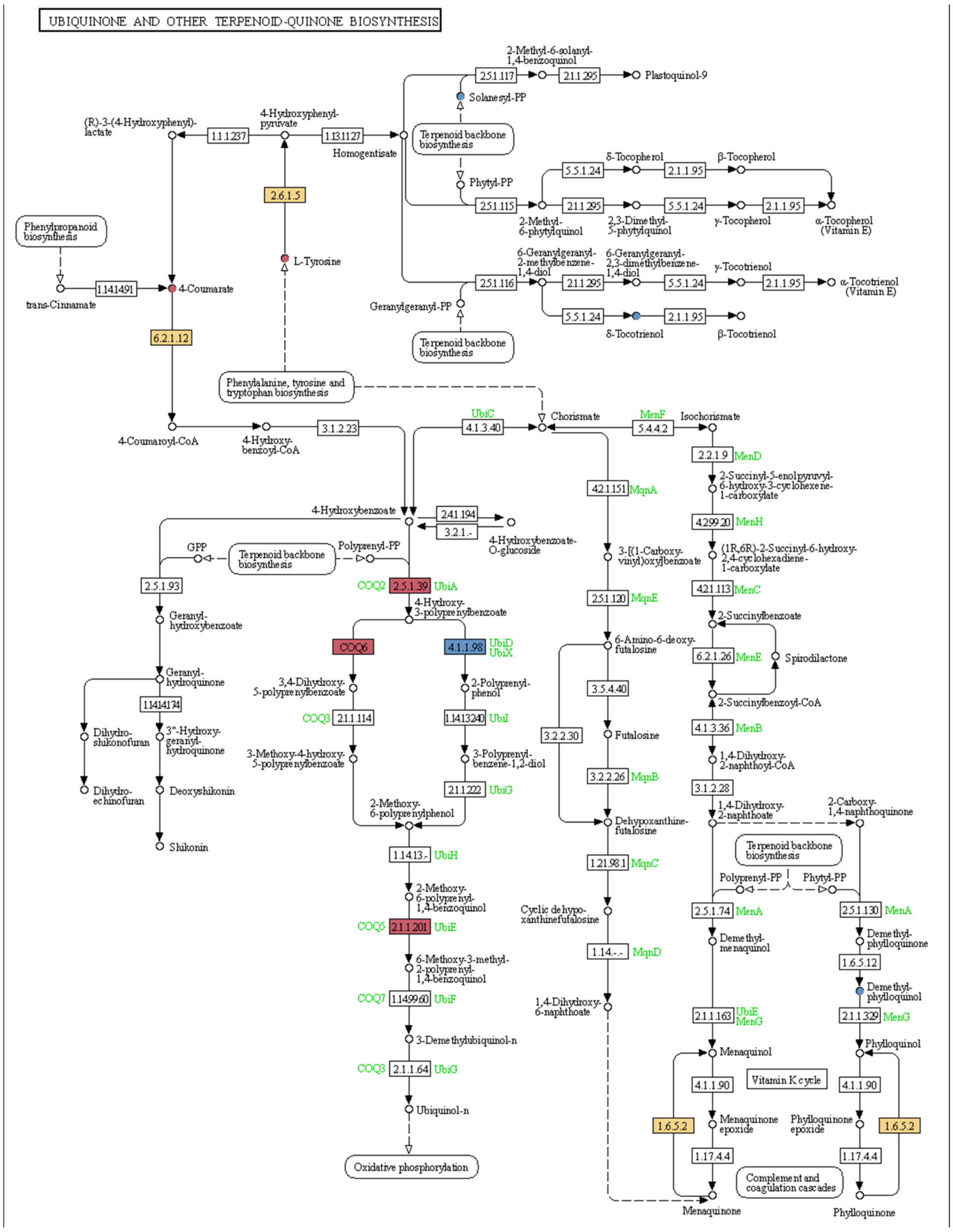

Figure S6. Ubiquinone and other terpenoid-quinone biosynthesis pathway.

In this pathway map, gene expression and metabolite changes are indicated by different colors and shapes: red rectangles represent upregulated genes ($log_2FC > 0$), blue rectangles represent downregulated genes ($log_2FC < 0$), and up- or down-regulated metabolites are shown as circles in the corresponding colors.

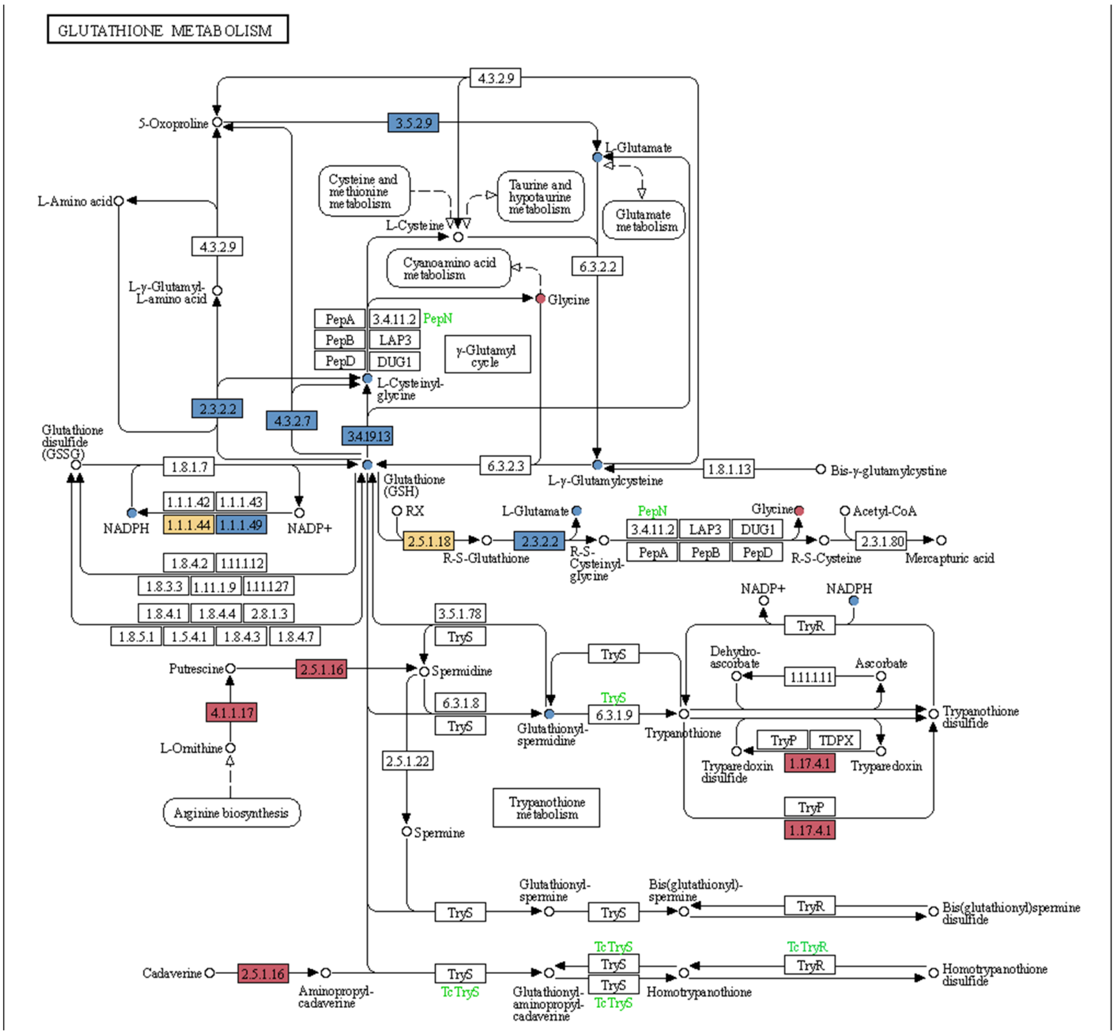

Figure S7. Glutathione metabolism pathway.

In this pathway map, gene expression and metabolite changes are indicated by different colors and shapes: red rectangles represent upregulated genes ($\log_2FC > 0$), blue rectangles represent downregulated genes ($\log_2FC < 0$), and up- or down-regulated metabolites are shown as circles in the corresponding colors.

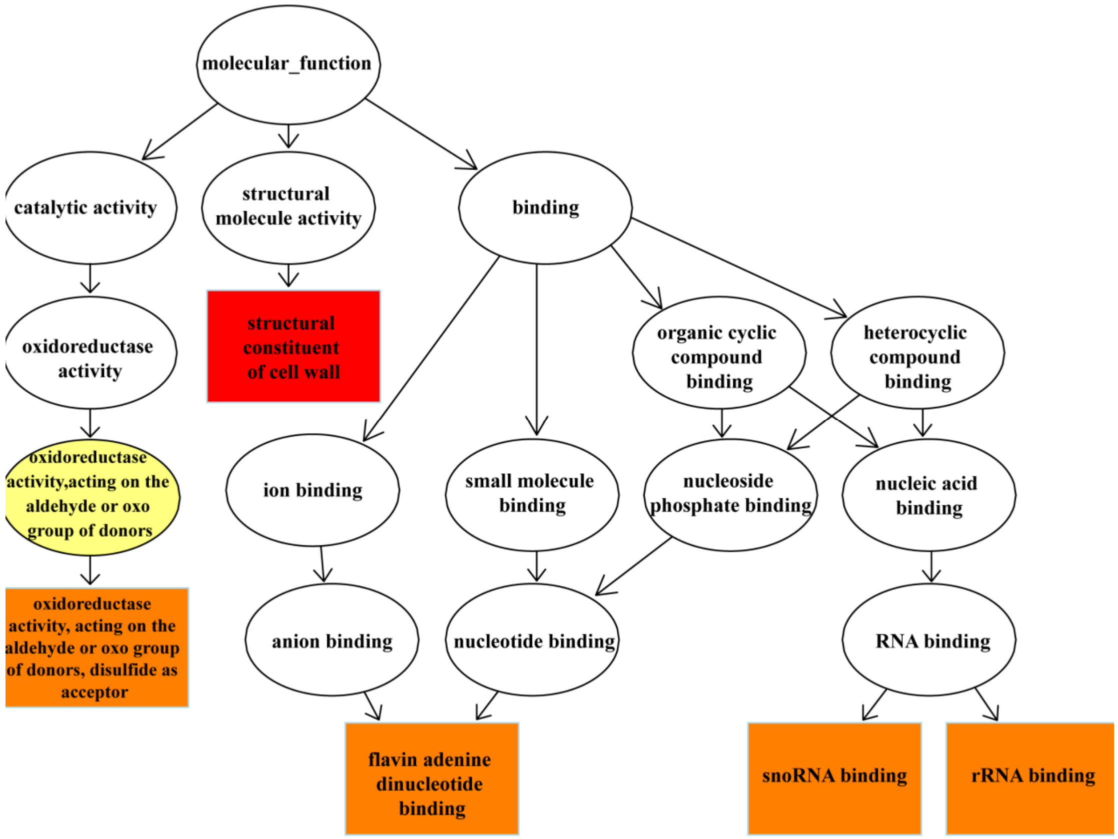

Figure S8. Directed acyclic graph of enriched Gene Ontology biological processes in *T. harzianum* under direct confrontation with *F. oxysporum*.

Each node represents a significantly enriched Gene Ontology biological process, and edges indicate hierarchical relationships among terms. Color intensity reflects the level of statistical significance, with white indicating no significant enrichment.

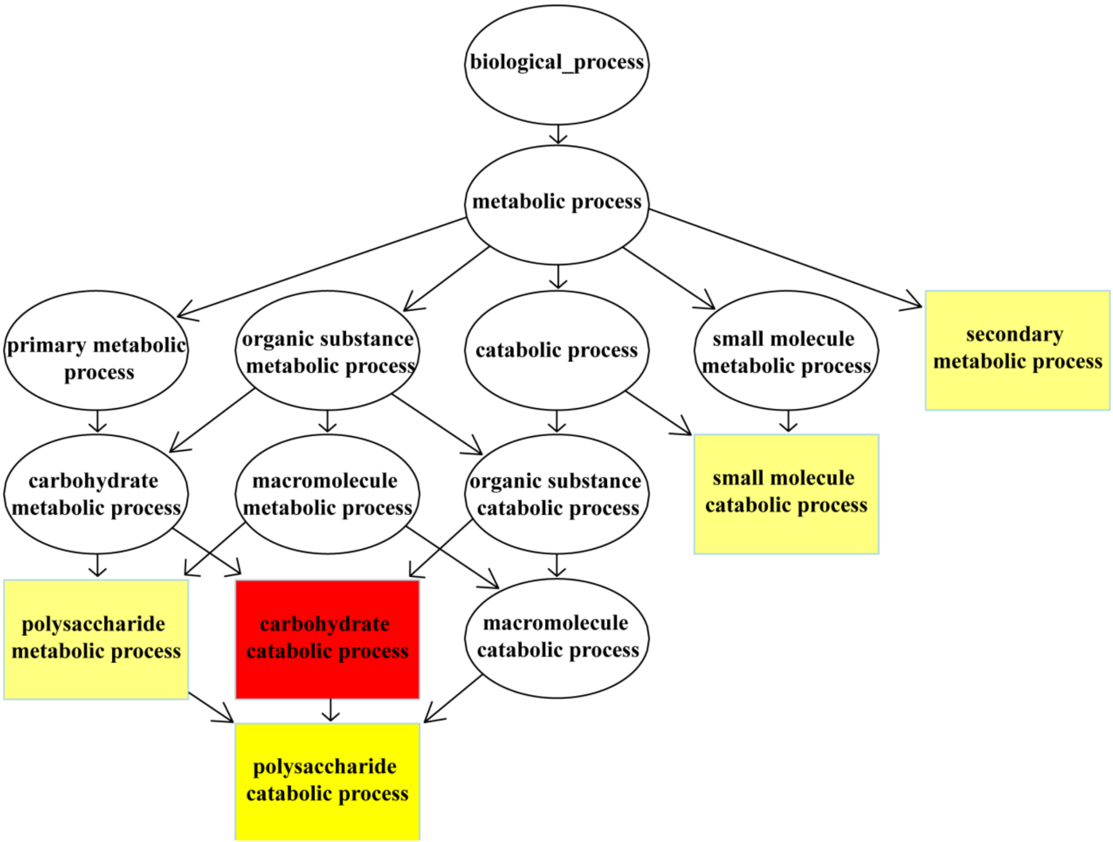

Figure S9. Directed acyclic graph of enriched Gene Ontology biological processes in *T. harzianum* under indirect confrontation with *F. oxysporum*.

Each node represents a significantly enriched Gene Ontology biological process, and edges indicate hierarchical relationships among terms. Color intensity reflects the level of statistical significance, with white indicating no significant enrichment.

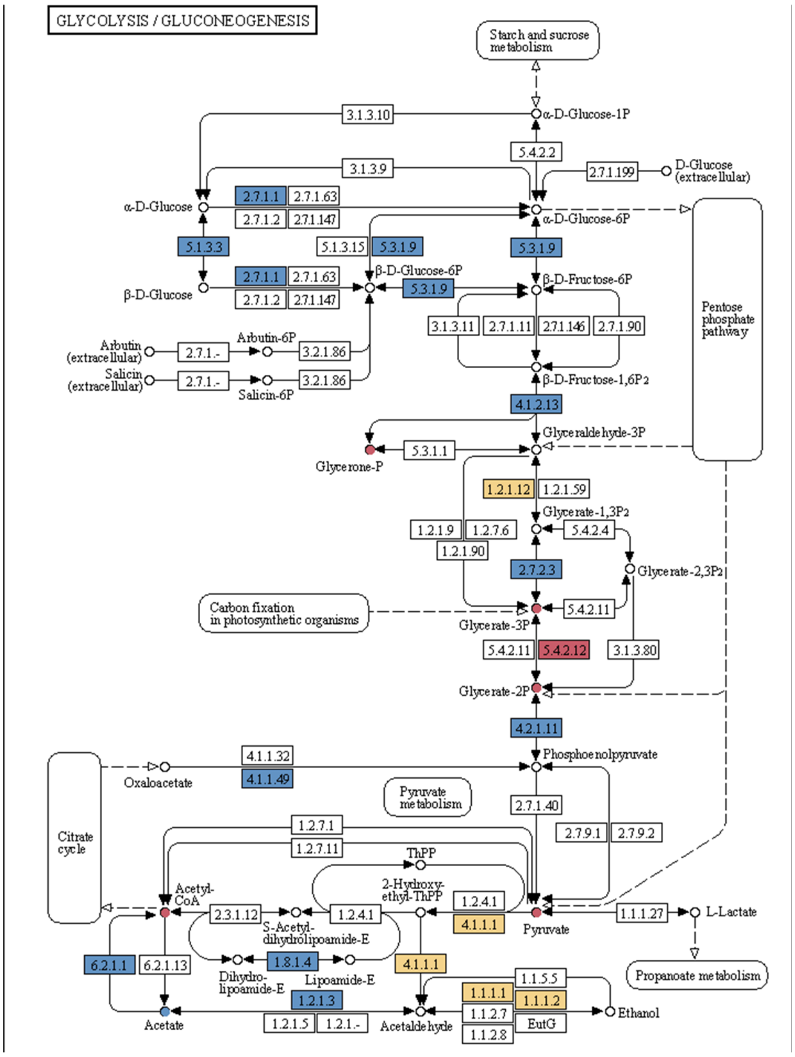

Figure S10. Glycolysis/Gluconeogenesis pathway.

In this pathway map, gene expression and metabolite changes are indicated by different colors and shapes: red rectangles represent upregulated genes ($\log_2FC > 0$), blue rectangles represent downregulated genes ($\log_2FC < 0$), and up- or down-regulated metabolites are shown as circles in the corresponding colors.

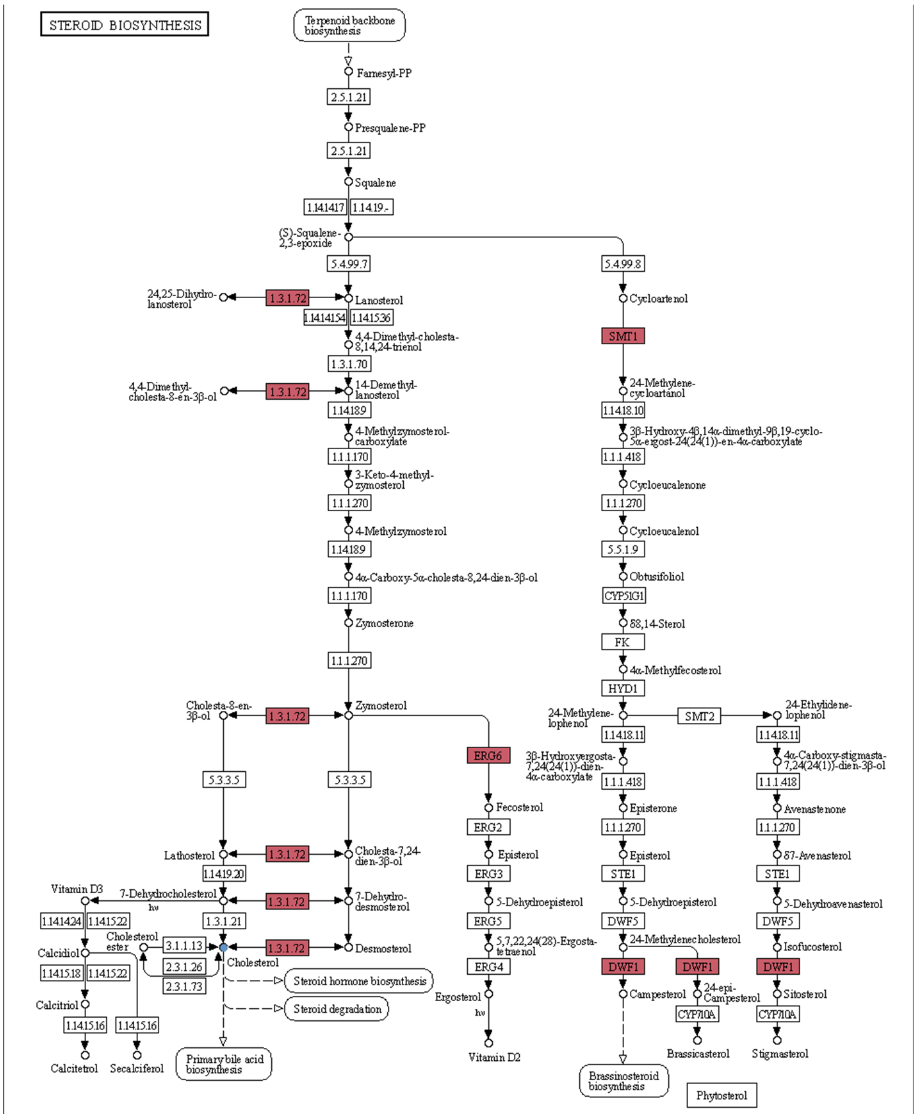

Figure S11. Steroid biosynthesis pathway.

In this pathway map, gene expression and metabolite changes are indicated by different colors and shapes: red rectangles represent upregulated genes ($\log_2FC > 0$), blue rectangles represent downregulated genes ($\log_2FC < 0$), and up- or down-regulated metabolites are shown as circles in the corresponding colors.

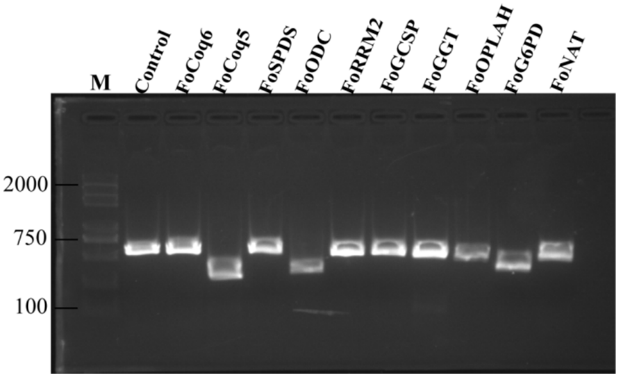

Figure S12. Agarose gel electrophoresis of chemically synthesized dsRNAs targeting *F. oxysporum* genes.

Table S8. Putative hypothetical protein genes screened in *T. harzianum* and their conserved domains.

Gene ID	Putative functional annotation	Conserved domains
M431DRAFT_141171	Sterol methyltransferase, potentially involved in sterol metabolism and signaling regulation	Sterol_MT_C; AdoMet_MTases
M431DRAFT_526980	Potentially involved in carbohydrate metabolism or the mevalonate pathway (HMG-CoA metabolism)	GlcD; PRK02304; P-mevalo_kinase
M431DRAFT_83118	Possibly associated with succinate–iron metabolism or lipid biosynthesis	Suc_Fer-like; LPLAT
M431DRAFT_515795	Potentially involved in glycerol-3-phosphate metabolism and redox reactions	Gp_dh_N
M431DRAFT_83452	Member of the hexokinase family, potentially affecting glucose metabolism	Hexokinase_1; Hexokinase_2
M431DRAFT_83864	Possibly involved in galactose metabolism and carbohydrate conversion	Galactose_mutarotase_like
M431DRAFT_497412	Involved in glycolysis and the pentose phosphate pathway	Glucose-6-phosphate isomerase
M431DRAFT_94438	Member of the fructose-bisphosphate aldolase family, potentially affecting carbohydrate metabolism	FBP_aldolase_IIA
M431DRAFT_510202	Involved in glycolysis, catalyzing the phosphorylation of phosphoglycerate	Phosphoglycerate kinase

Table S9. A260 and A280 absorbance values and concentrations of dsRNA obtained using different extraction methods.

Extraction method	A260	A280	Concentration (ng·μL^{-1})
cell suspension	55.77±1.71	24.19±0.50	2230.93±68.57
TRIzol	54.86±0.71	23.82±0.46	2194.40±28.26
